# A Unified Damage–Plasticity Constitutive Framework for Freeze–Thaw-Damaged Concrete Under Monotonic and Cyclic Compression

**DOI:** 10.3390/ma19173740

**Published:** 2026-09-02

**Authors:** Ping Gao, Wenlong Zhao, Jinbo Xie, Xi Du, Yungui Pan, Lixin Chang

**Affiliations:** 1 School of Urban Regeneration, Shanghai Zhongqiao Vocational and Technical University, Shanghai 201514, China; 2 Department of Civil Engineering, School of Environment and Architecture, University of Shanghai for Science and Technology, Shanghai 200093, China; 3 Department of Engineering Geology, Institute of Applied Geosciences, Technical University of Berlin, Ernst-Reuter-Platz 1, 10587 Berlin, Germany; 4 College of Intelligent Building Engineering, Shanghai Construction Management Vocational College, Shanghai 201702, China

**Keywords:** freeze–thaw damage, concrete, damage–plasticity model, cyclic compression, residual strain, cross-dataset evaluation

## Abstract

To provide a unified description of the monotonic and cyclic compressive responses of concrete after freeze–thaw exposure, a one-dimensional phenomenological damage–plasticity constitutive framework is proposed. Freeze–thaw-induced pre-damage is quantified by the degradation of the initial static stiffness. A Mander-type equation is employed to describe the monotonic envelope, while residual strain is introduced to characterize plastic deformation. A signed stiffness variable is defined to distinguish pre-peak compaction from post-peak mechanical damage, and the unloading and reloading paths are represented by piecewise power-law functions for the pre-peak and post-peak regimes. The model is evaluated using 36 monotonic compression curves of recycled coarse aggregate self-compacting concrete subjected to sulfate freeze–thaw cycles and cyclic compression data for ordinary concrete subjected to seawater freeze–thaw cycles. After independent identification of the envelope-shape parameters, the monotonic responses yield an average *R*^2^ of 0.978 and an average NRMSE of 0.041, whereas the complete cyclic responses yield an average *R*^2^ of 0.941 and an average NRMSE of 0.066. Because the model parameters are identified separately using data from each freeze–thaw exposure level, these accuracy measures characterize parameter calibration and response reconstruction rather than independent prediction of untested exposure states. Freeze–thaw exposure causes substantially greater degradation of the initial static elastic modulus than reduction in peak stress, indicating that stiffness, strength, peak strain, and envelope shape should be treated as state variables at different hierarchical levels. The cyclic unloading stiffness exhibits both pre-peak enhancement and post-peak degradation, confirming the necessity of separately representing compaction and mechanical damage. The proposed model provides a unified representation of the monotonic envelope, residual deformation, and principal cyclic hysteretic characteristics of freeze–thaw-damaged concrete.

## 1. Introduction

Freeze–thaw cycling is a major factor affecting the durability and service safety of concrete structures in cold regions. When pore water repeatedly freezes and thaws, the material undergoes ice-crystal growth, moisture migration, and the associated cyclic local pressures, which progressively induce pore expansion, interfacial weakening, and microcrack accumulation. Freeze–thaw exposure is generally manifested by reductions in the static elastic modulus and strength, changes in peak strain, and alterations in the overall shape of the stress–strain curve [1,2,3,4,5]. For bridges, ports, offshore platforms, and transportation infrastructure in cold regions, conventional indicators such as mass loss, relative dynamic elastic modulus, or single-point compressive strength cannot directly support nonlinear structural analysis. Establishing a constitutive relationship capable of describing the complete deformation process, damage evolution, and cyclic loading paths of concrete after freeze–thaw exposure is therefore essential for linking material deterioration to structural response.

Actual service environments rarely involve freeze–thaw cycling in freshwater alone. Seawater and deicing-salt environments introduce chlorides and other dissolved constituents, whereas sulfate environments may additionally involve salt crystallization and chemical reactions, resulting in interactions between freeze–thaw-induced physical damage and chemical attack [6,7,8,9,10]. Meanwhile, recycled coarse aggregate concrete and recycled coarse aggregate self-compacting concrete have the potential to reduce the consumption of natural aggregates and the landfilling of construction and demolition waste through the reuse of waste-concrete aggregates. However, adhered old mortar, pre-existing cracks, and multiple interfacial transition zones make their freeze–thaw durability and mechanical response more strongly dependent on material composition [7,8,9,10,11]. A constitutive framework applicable to different material compositions and salt–freeze environments must therefore retain a unified mechanical structure without using a single empirical damage variable to represent all state parameters.

Existing studies on the monotonic compressive behavior of freeze–thaw-damaged concrete have mainly focused on the degradation of peak parameters, complete stress–strain relationships, and environmental damage variables [1,2,3,4,5]. Mander-type formulations have also been adopted to describe the complete monotonic envelope using a limited number of parameters [12,13], while statistical approaches such as Weibull models have been used to characterize macroscopic freeze–thaw deterioration [8]. These studies demonstrate that freeze–thaw effects can be represented through state-dependent parameters; however, the experimentally measured static elastic modulus, peak-response parameters, and mathematical envelope-shape parameters are not necessarily equivalent state descriptors. In particular, directly equating the measured static modulus with the initial tangent implied by an envelope equation may couple experimentally measured stiffness degradation with the mathematical parameter controlling the complete curve shape.

Research on the cyclic compressive behavior of ordinary concrete has provided a foundation for describing loading-history effects, permanent deformation, and hysteretic paths. Classical cyclic concrete models have established the basis for describing residual deformation, unloading–reloading behavior, stiffness degradation, and damage evolution under repeated compression [14,15,16,17,18,19]. Beyond pointwise stress–strain reconstruction, energy dissipation provides an additional perspective for evaluating cyclic concrete behavior. Scerrato et al. [20] experimentally investigated the internal dissipation of concrete under cyclic loading and used energy-related response characteristics together with parameter identification to assess dissipative behavior. Although their study concerned enriched concrete rather than freeze–thaw-damaged concrete, it illustrates the broader importance of considering dissipation measures in addition to local stress–strain agreement. In the present study, hysteretic energy is therefore introduced as an independent mechanical diagnostic of the reconstructed cyclic paths rather than as an additional fitted parameter.

For freeze–thaw-damaged concrete, Qiu et al. [13] and subsequent studies [6,21] extended cyclic constitutive descriptions to specimens subjected to different freeze–thaw exposure conditions. However, freeze–thaw exposure changes the initial stiffness baseline, peak-response scale, and defect state before mechanical loading begins, making direct transfer of models developed for undamaged concrete problematic without recalibration. Compared with the model of Qiu et al. [13], which combines a modified Mander envelope with plastic-damage theory for concrete subjected to seawater freeze–thaw exposure, the present framework differs primarily in the organization and interpretation of its state variables. The experimentally measured static stiffness is separated from the equivalent initial tangent stiffness of the envelope; freeze–thaw pre-damage is distinguished from subsequent mechanically induced stiffness changes; a signed stiffness variable is introduced to represent pre-peak stiffness enhancement without invoking negative damage; and the envelope, residual-strain, and cyclic-path parameters are identified hierarchically. Hysteretic energy is further used as an independent mechanical diagnostic rather than as an additional fitting target. Thus, the principal contribution of the present study lies in the hierarchical integration and state-level separation of these constitutive components for sequential freeze–thaw and mechanical loading.

Nevertheless, several common issues remain in existing cyclic models. First, freeze–thaw damage is generally treated as initial damage, whereas subsequent mechanical damage is determined from the degradation of unloading stiffness. Clear state definitions are required to couple these two damage mechanisms while avoiding duplicated reductions in stiffness or strength. Second, the pre-peak unloading stiffness may exceed the initial stiffness measured after freeze–thaw exposure. Directly defining damage as (1 − *E*/*E*_0_), where *E* is the unloading stiffness and *E*_0_ is the initial stiffness, would therefore produce a negative damage value. Such stiffness enhancement is consistent with mechanisms such as pore compaction, closure of pre-existing microcracks, and progressive engagement of contact interfaces in load transfer; however, these mechanisms are not independently verified by the macroscopic data used in the present study. Third, satisfactory pointwise fitting of cyclic stress–strain curves does not guarantee the unique identification of residual strain, unloading stiffness, damage variables, and hysteretic energy dissipation. In particular, stiffness calculated from coordinate differences and energy obtained by path integration are more sensitive to experimental resolution and path segmentation.

The integration of recycled coarse aggregates with self-compacting concrete technology provides a material pathway for waste-concrete valorization and the construction of geometrically complex structural components. However, the water-absorption characteristics of recycled coarse aggregates, adhered old mortar, and multiple-interface structure can alter transport properties and freeze–thaw responses. Tuyan et al. [11] investigated the mechanical, transport, and freeze–thaw properties of self-compacting concrete containing coarse recycled aggregates and showed that the incorporation of recycled coarse aggregate significantly affected freeze–thaw durability. Xiao et al. [7] examined the effects of sulfate concentration and recycled coarse aggregate replacement ratio on the freeze–thaw deterioration of recycled concrete and developed damage models based on mass, dynamic elastic modulus, strength, and microscopic reaction products.

Recent studies have further extended the research object to recycled coarse aggregate self-compacting concrete. Zheng et al. [9] investigated the effects of recycled coarse aggregate replacement ratio, sulfate environment, and number of freeze–thaw cycles on the durability indices of recycled coarse aggregate self-compacting concrete. The same research group subsequently obtained complete uniaxial stress–strain curves and developed both empirical models and machine-learning-based predictive models [10]. These studies have substantially enriched the macroscopic database covering different mixture proportions and salt–freeze environments. Nevertheless, material durability indices, monotonic compressive envelopes, and cyclic damage variables are still commonly treated separately in different studies. Moreover, a continuous parameter-evolution law applicable to arbitrary mixture proportions and exposure media cannot be established solely from one nonzero salt concentration or a limited number of recycled coarse aggregate replacement levels.

Overall, substantial progress has been made in the monotonic and cyclic constitutive modeling of concrete after freeze–thaw exposure. However, a unified framework that strictly corresponds to experimentally identifiable quantities and clearly distinguishes different levels of evidence remains lacking. Accordingly, the present study focuses on the following issues.

Based on the above review, the principal research gaps addressed in this study are as follows.

(1) Freeze–thaw-induced pre-damage and mechanically induced damage are not always clearly separated. Because initial stiffness, peak stress, peak strain, and envelope shape exhibit different sensitivities to freeze–thaw exposure, they should not be represented indiscriminately by a single damage variable.
(2) The experimentally measured static stiffness is not necessarily identical to the equivalent initial tangent stiffness implied by a complete envelope equation. Their roles in freeze–thaw damage characterization and envelope-shape identification therefore need to be distinguished.
(3) Pre-peak stiffness enhancement and post-peak stiffness degradation require different state interpretations. Treating both through a conventional non-negative damage variable may incorrectly represent compaction-induced stiffness recovery as negative damage.
(4) Simultaneous calibration of envelope, residual-strain, and cyclic-path parameters may allow different sources of fitting error to compensate for one another. A hierarchical identification strategy is therefore required to improve parameter interpretability.
(5) More generally, constitutive-model assessment should distinguish uncertainty associated with experimental data. Monotonic data cannot validate cyclic internal variables, and a high *R*^2^ value obtained from data at the same exposure level does not demonstrate blind predictive capability across different freeze–thaw exposure levels. A hierarchical evidence chain should therefore be established using datasets involving different material types, exposure media, and loading paths. Hysteretic energy dissipation should also be treated as an independent mechanical diagnostic rather than evaluating the model solely on the basis of pointwise stress errors. More generally, constitutive-model assessment should distinguish uncertainty associated with experimental data and parameter identification from uncertainty associated with the model representation and its predictive use. Probabilistic uncertainty-quantification frameworks provide systematic approaches for addressing these different sources of uncertainty [22]; however, the datasets available in the present study do not contain the replicate-level observations or original acquisition records required for such an analysis. The present evaluation is therefore deliberately restricted to deterministic parameter identification, response reconstruction, and explicit reporting of the corresponding evidence and uncertainty boundaries.

To address these issues, this study develops a one-dimensional, envelope-driven damage–plasticity constitutive framework for concrete subjected to a sequential “freeze–thaw exposure followed by mechanical loading” history. The proposed framework is evaluated hierarchically using two published datasets covering monotonic and cyclic compression responses under different freeze–thaw environments. The datasets are used to examine the consistency of the constitutive architecture after recalibration rather than to claim universal transferability of the calibrated parameters. It should be emphasized that the individual mathematical ingredients adopted in this framework, including the Mander-type envelope, residual-strain description, and unloading–reloading functions, are based on established constitutive concepts. The contribution of the present study lies primarily in their hierarchical integration and state-level separation for the sequential freeze–thaw–mechanical loading problem, rather than in claiming each individual formulation as a new constitutive theory.

The main contributions of this study are summarized as follows: (1) a one-dimensional framework is established in which the freeze–thaw state, monotonic envelope, residual strain, and cyclic paths are represented at distinct hierarchical levels, thereby avoiding the use of a single damage variable to replace all mechanical characteristics; (2) the experimentally measured static modulus is explicitly distinguished from the equivalent tangent stiffness of the Mander envelope, such that $E_{N}$ is used to quantify freeze–thaw pre-damage, whereas $r_{N}$ is reserved exclusively for controlling the envelope shape; (3) a signed stiffness variable is introduced to represent pre-peak stiffness enhancement as an operational compaction variable and post-peak stiffness degradation as non-negative mechanical damage, thereby avoiding the interpretation of stiffness enhancement as negative damage; (4) a hierarchical identification strategy is adopted for the envelope, residual strain, and path parameters, together with a two-level data-evaluation procedure that simultaneously reports evidence from stress reconstruction, internal-variable consistency, and hysteretic energy dissipation; and (5) the scope of applicability and predictive boundaries of the model are explicitly defined, with the present conclusions restricted to parameter identification, response reconstruction, and consistency of the constitutive architecture.

Accordingly, the objectives of this study are to: (1) establish a hierarchical constitutive framework that separates freeze–thaw pre-damage, pre-peak compaction, and post-peak mechanical damage; (2) distinguish experimentally measured static stiffness from the equivalent tangent stiffness of the monotonic envelope and identify the model parameters hierarchically; and (3) evaluate the framework using monotonic and cyclic responses in terms of stress reconstruction, internal-variable consistency, and hysteretic energy dissipation while explicitly defining its validation and predictive boundaries.

## 2. Damage–Plasticity Constitutive Framework for Freeze–Thaw-Pre-Damaged Concrete Under Uniaxial Cyclic Compression

This section establishes a one-dimensional phenomenological constitutive framework for the sequential loading history of freeze–thaw exposure followed by mechanical loading. The state of the specimen after completion of the freeze–thaw treatment is adopted as the reference configuration for the subsequent mechanical loading stage, with compressive stress and compressive strain defined as positive. The environmental effects are incorporated through the initial static stiffness, peak stress, peak strain, and envelope-shape parameters, whereas the mechanical loading response is characterized by residual strain, pre-peak compaction, post-peak damage, and the unloading–reloading paths. The framework is formulated for small-strain, isothermal, and quasi-static uniaxial compression and does not involve a multiaxial yield surface, plastic potential, or consistency condition [18,23,24]. The plastic strain introduced herein is an identifiable one-dimensional internal variable defined from the residual strain measured at zero stress [19]. Accordingly, the present formulation should be regarded as a uniaxial phenomenological material-point constitutive framework rather than as a general three-dimensional damage–plasticity model for direct structural analysis.

### 2.1. Reference Configuration, Strain Decomposition, and History Variables

Freeze–thaw cycling may induce pore expansion, interfacial weakening, and microcracking within the material, together with free expansion or contraction. Because the post-freeze–thaw state is taken as the zero-strain reference for the subsequent mechanical test, the free deformation accumulated during freeze–thaw exposure is not explicitly included in the axial strain decomposition. Instead, its effects are represented collectively by the freeze–thaw pre-damage variable and the state parameters corresponding to the specified exposure level [1,2,3,4,12]. The total strain is decomposed into a recoverable component and a zero-stress residual component:

(1) $$\varepsilon =\varepsilon^{e}+\varepsilon^{p}$$

where ε is the total axial strain, $\varepsilon^{e}$ is the recoverable strain, and $\varepsilon^{p}$ is the residual strain retained after unloading to zero stress. This decomposition is introduced to distinguish stiffness variation from permanent deformation; it does not imply that a complete three-dimensional plastic-flow theory is established in the present study.

The loading history is characterized by the maximum historical strain *k*, which is normalized by the peak strain $\varepsilon_{e}$, corresponding to the specified freeze–thaw exposure level:

(2) $$\kappa \left(t\right)=\underset{\tau \leq t}{max}\;\varepsilon \left(\tau \right),\eta =\frac{\kappa }{\varepsilon_{c,N}}$$

where η the normalized historical maximum strain; During monotonic loading or continued loading beyond the previously attained maximum strain, κ = ε. During unloading and reloading that does not exceed the current value of κ, the history variable κ remains unchanged. To avoid confusion with the coding of material groups, the number of freeze–thaw cycles is consistently denoted by $N_{FT}$.

### 2.2. Freeze–Thaw Pre-Damage and Cumulative Damage Index

Let $E_{0}$ denote the initial static stiffness of concrete without freeze–thaw exposure, and let $E_{N}$ denote the initial static stiffness after $N_{FT}$ freeze–thaw cycles. The stiffness retention ratio is used to characterize the reduction in the initial load-bearing capacity caused by environmental exposure, and the freeze–thaw pre-damage variable is defined as:

(3) $$d_{f}\left(N_{FT}\right)=1-\frac{E_{N}}{E_{0}},\;E_{N}=\left(1-d_{f}\right)E_{0}$$

A consistent definition of static stiffness must be adopted for $E_{N}$ across all experimental groups. This parameter is used to identify freeze–thaw pre-damage and to evaluate the cyclic unloading stiffness and must not be conflated with the equivalent initial tangent derived from the envelope function. The relative dynamic elastic modulus may be used as a durability indicator, but it can replace the static stiffness $E_{N}$ only when an explicit conversion relationship has been established.

The mechanical damage variable *d_m_* represents the additional degradation of load-bearing capacity induced by mechanical loading relative to the post-freeze–thaw reference configuration. If both damage components are interpreted in terms of the effective load-bearing fraction, the total effective fraction is expressed as the product of their respective retention factors:

(4) $$1-D=\left(1-d_{f}\right)\left(1-d_{m}\right),\;D=d_{f}+d_{m}-d_{f}d_{m}$$

where $d_{f}$ is the freeze–thaw pre-damage variable. The variable *D* in Equation (4) is a cumulative index combining freeze–thaw pre-damage and mechanically induced damage, with the interaction term preventing double counting of the two damage contributions. *D* is not introduced into the stress calculation as an additional independent parameter, thereby avoiding duplicated degradation already represented by $E_{N}$ and $d_{m}$. The pre-peak compaction variable $c_{m}$ characterizes the stiffness enhancement associated with defect closure. Because this mechanism does not represent an irreversible loss of the effective load-bearing area, $c_{m}$ is not included in D.

It should be emphasized that they are operational material-point state variables defined within the present framework rather than directly measured fractions of physical damage. The variable quantifies the reduction in initial static stiffness caused by freeze–thaw exposure relative to the unfrozen reference state, whereas characterizes the additional mechanically induced stiffness degradation relative to the corresponding post-freeze–thaw reference state. The cumulative index provides a retention-based representation of their combined effect while avoiding arithmetic double counting. It is a derived diagnostic quantity and is not introduced as an independent stress-calculation parameter.

### 2.3. Monotonic Envelope Submodel

The uniaxial compressive envelope of concrete after freeze–thaw exposure generally comprises an initial ascending stage, pre-peak nonlinearity, the peak response, and post-peak softening. In this study, a Mander-type single-equation formulation with a limited number of parameters is adopted to represent the envelope [12,15]:

(5) $$\sigma_{env}=f_{c,N}\frac{r_{N}x}{r_{N}-1+x^{r_{N}}},\;x=\frac{\kappa }{\varepsilon_{c,N}}$$

where $\sigma_{env}$ is the compressive stress on the monotonic outer envelope corresponding to the current historical maximum strain; $x^{r_{N}}$ denotes the $r_{N}th$ power of the normalized historical maximum strain. $f_{c,N}$ and $\varepsilon_{c,N}$ are the peak stress and peak strain, respectively, after $N_{FT}$ freeze–thaw cycles, and ($r_{N}$ > 1) is the envelope-shape parameter. The function automatically passes through the origin and the peak point and attains its maximum at (*x* = 1). The equivalent tangent stiffness at the origin is given by:

(6) $$E_{env,N}=\underset{\varepsilon \to 0}{lim}\;\frac{\sigma_{env}}{\varepsilon }=\frac{r_{N}}{r_{N}-1}\frac{f_{c,N}}{\varepsilon_{c,N}}$$

The equivalent initial tangent $E_{env,N}$ is determined by the overall shape of the envelope function and is generally not equal to the static stiffness $E_{N}$ measured over a prescribed stress interval. $\varepsilon_{c,N}$ is the axial compressive strain corresponding to the peak stress after $N_{FT}$ freeze–thaw cycles, i.e., the peak strain at the specified freeze–thaw exposure level. Accordingly, $f_{c,N}$ and $\varepsilon_{c,N}$ are determined directly from the experimental peak response, whereas $r_{N}$ is identified independently from the complete envelope curve. Section 3 evaluates this submodel and the proposed parameter-separation strategy using monotonic compression data for recycled coarse aggregate self-compacting concrete subjected to sulfate freeze–thaw exposure. Monotonic curves alone cannot independently identify $\varepsilon^{p}$, $c_{m}$ and $d_{m}$.

### 2.4. Evolution of Plastic Residual Strain

During cyclic compression of concrete, the unloading path generally does not return to the strain origin, and the residual strain increases with the historical maximum strain. Based on the classical quadratic relationship for residual strain, while allowing freeze–thaw exposure to modify its magnitude and curvature, the following expression is adopted:

(7) $$\frac{\varepsilon^{p}}{\varepsilon_{c,N}}=A_{N}\eta^{2}+B_{N}\eta$$

The parameters $A_{N}$ and $B_{N}$ are identified from the unloading initiation points and the residual-strain points measured at low stress for each freeze–thaw exposure level. Within the calibration range, the condition ($0\leq e^{p}<k$) must be satisfied. Unconstrained extrapolation is not permitted beyond the experimentally covered range of η. Equation (7) determines the position of the hysteresis loop along the strain axis and is identified hierarchically and separately from the parameters governing the curvature of the unloading–reloading paths.

### 2.5. Pre-Peak Compaction, Post-Peak Damage, and State Relationships

If all changes in unloading stiffness are interpreted as damage, the pre-peak stage may exhibit ($E_{u}>E_{n}$), resulting in so-called “negative damage.” For example, the minimum identified signed stiffness variable was, corresponding to an unloading stiffness of approximately. If the same state were evaluated using the conventional stiffness-based definition, the resulting damage value would be. In the present formulation, this stiffness enhancement is instead assigned to the non-negative compaction variable, thereby avoiding the interpretation of stiffness recovery as negative mechanical damage. Cyclic tests indicate that this phenomenon occurs systematically in concrete after freeze–thaw exposure and is more reasonably attributed to pore closure, the compaction of pre-existing cracks, and the progressive engagement of contact interfaces in load transfer [13,16,17]. For the *i*-th cycle, the experimental unloading secant stiffness and the signed stiffness variable are defined as:

(8) $$E_{u,i}=\frac{\sigma_{u,i}}{\kappa_{i}-\varepsilon_{i}^{p}},\;\omega_{i}^{obs}=1-\frac{E_{u,i}}{E_{n}}$$

For a state on the envelope, the corresponding signed stiffness variable can also be calculated from the envelope stress and residual strain:

(9) $$\omega^{cal}\left(\kappa \right)=1-\frac{\sigma_{env}\left(\kappa \right)}{E_{h}\left[\kappa -\varepsilon^{\rho }\left(\kappa \right)\right]}$$

A negative value of ω indicates stiffness enhancement relative to $E_{n}$, whereas a positive value indicates stiffness degradation. The positive-part operator is employed to decompose ω into the compaction variable $d_{m}$ and the non-negative mechanical damage variable $c_{m}$:

(10) $$c_{m}=\left\langle -\omega \right\rangle ,\;d_{m}=\left\langle \omega \right\rangle ,\;\left\langle z\right\rangle =max\left(z,0\right)$$

The corresponding one-dimensional state relationship is written as:

(11) $$\sigma =\left(1+c_{m}-d_{m}\right)E_{N}\left(\varepsilon -\varepsilon^{p}\right)$$

Using ($E_{N}=\left(1-d_{f}\right)E_{0}$ and Equations (4) and (11) can also be rewritten as:

(12) $$\sigma =\left[\left(1-D\right)+\left(1-d_{f}\right)c_{m}\right]E_{0}\left(\varepsilon -\varepsilon^{p}\right)$$

Equation (12) indicates that the cumulative damage index accounts only for the reduction in load-bearing capacity caused by freeze–thaw exposure and mechanical damage, whereas the compaction contribution appears separately through the term $\left(1-d_{f}\right)c_{m}$. During envelope loading, the stress is generated directly using Equation (5), while Equations (9)–(12) are employed to interpret and verify the internal variables. The proposed model is therefore a one-dimensional framework that is envelope-driven, with the internal variables serving an interpretative role.

### 2.6. Unloading–Reloading Paths

Let the *i*-th cycle begin unloading from the envelope point ($\kappa_{i}$, $\sigma_{u,i}$), with the corresponding residual Fstrain denoted by $\varepsilon_{i}^{p}$. The normalized recoverable strain χ is defined as:

(13) $$\chi =\frac{\varepsilon -\varepsilon_{i}^{p}}{\kappa_{i}-\varepsilon_{i}^{p}},\;0\leq \chi \leq 1$$

The curvatures of the pre-peak and post-peak cyclic paths differ. Accordingly, s=pre is assigned when $\eta_{i}\leq 1$, whereas s=post is assigned when $\eta_{i}>1$. The unloading and reloading branches are then described separately using power-law functions:

(14) $$\sigma_{un}=\sigma_{u,i}\chi^{n_{u,N}^{s}},\;s\in \left\{pre,post\right\}$$

(15) $$\sigma_{re}=\sigma_{u,i}\chi^{n_{r,N}^{s}},\;s\in \left\{pre,post\right\}$$

where $\chi^{n_{u,N}^{s}}$ is the dimensionless shape exponent of the unloading path; $\chi^{n_{r,N}^{s}}$ is the dimensionless shape exponent of the reloading path. All path exponents are constrained to be positive. At χ=0, both branches pass through the residual point, whereas at χ=1, they return to the historical maximum-strain point. When a path exponent equals unity, the corresponding branch reduces to a linear path. During unloading and reloading, κ, $\varepsilon^{p}$, $c_{m}$, and $d_{m}$ remain fixed. Once the reloading strain exceeds $\kappa_{i}$, the response returns to the outer envelope:

(16) $$\varepsilon >\kappa_{i}:\;\kappa \leftarrow \varepsilon ,\;\sigma =\sigma_{env}\left(\varepsilon \right)$$

This switching rule distinguishes between returning to the previous historical maximum strain and continued envelope loading after that maximum strain has been exceeded, thereby preventing the subsequent envelope response from being incorrectly treated as part of the same reloading branch [10,14].

### 2.7. Cyclic Energy Dissipation and Mechanical Admissibility

Curve-fitting accuracy cannot substitute for fundamental mechanical constraints. For Equations (14) and (15), the area enclosed by the *i*-th closed hysteresis loop can be expressed analytically as:

(17) $$W_{h,i}=\sigma_{u,i}\left(\kappa_{i}-\varepsilon_{i}^{p}\right)\left|\frac{1}{n_{u,N}^{s}+1}-\frac{1}{n_{r,N}^{s}+1}\right|$$

$W_{h,i}$ denotes the magnitude of hysteretic energy dissipation and must be non-negative. Because positive-exponent power functions do not intersect within 0<χ<1, the relative magnitudes of $n_{u,N}^{s}$ and $n_{r,N}^{s}$ determine the vertical ordering of the unloading and reloading branches. The sign of the corresponding signed integral, prior to taking its absolute value, should be consistent with the actual traversal direction of the experimental loop. Therefore, in addition to requiring positive path exponents, parameter identification should verify that: (1) the unloading and reloading branches do not exhibit non-physical intersections within the loop; (2) the calculated hysteresis-loop area is consistent with the experimentally observed ordering of the two branches; and (3) the areas of the early small loops are not dominated by minor digitization errors. In the present study, $W_{h,i}$ is used as a diagnostic measure of mechanical admissibility rather than as an independently fitted parameter.

In addition, the model parameters must satisfy $0\leq d_{f}<1$, $0\leq d_{m}<1$, $c_{m}\geq 0$ and $0\leq \varepsilon^{p}<\kappa$. The continuous envelope should remain non-negative throughout the post-peak regime until the stress approaches zero. If the experimental response exhibits an abrupt stress drop, this behavior is treated as a boundary of applicability of the continuous phenomenological model, rather than being forcibly reproduced through anomalous parameter values intended to track localized instability.

### 2.8. Hierarchical Parameter Identification and Numerical Implementation

The model parameters are identified hierarchically to prevent mutual compensation arising from the simultaneous unconstrained optimization of all parameters. At the first level, $f_{c,N}$, $\varepsilon_{c,N}$, and $r_{N}$ are determined from the monotonic envelope or the outer envelope extracted from the cyclic response. At the second level, $A_{N}$ and $B_{N}$ are determined from the unloading initiation points and residual-strain points. At the third level, the unloading and reloading exponents for the pre-peak and post-peak regimes are identified separately in normalized coordinates.

The objective function used to identify the envelope-shape parameter is defined as:

(18) $$r_{N}=\arg\underset{r>1}{min}\;\overset{n}{\underset{j=1}{\sum {\left[\sigma_{env}\left(\varepsilon_{j};r\right)-\sigma_{j}^{exp}\right]}^{2}}}$$

The residual-strain parameters are identified by minimizing the following objective function:

(19) $$\left(A_{N},B_{N}\right)=\arg min\overset{M}{\underset{i=1}{\sum {\left[\varepsilon_{i}^{p,cal}-\varepsilon_{i}^{p,exp}\right]}^{2}}}$$

To prevent an individual cycle containing a larger number of data points from dominating the identification of the path parameters, each hysteretic branch is assigned equal weight in the objective function:

(20) $$n^{s}=\arg\underset{n>0}{min}\;\frac{1}{M_{s}}\sum_{i=1}^{M_{s}}\;\frac{1}{m_{i}}\overset{m_{i}}{\underset{j=1}{\sum {\left[{\hat{\sigma }}_{ij}^{cal}\left(n\right)-{\hat{\sigma }}_{ij}^{exp}\right]}^{2}}}$$

where $M_{s}$ is the number of cycles in regime s, $m_{i}$ is the number of data points on the *i*-th branch, and the hatted stresses denote quantities normalized by the stress at the corresponding branch endpoint. After parameter identification, the residual-strain bounds, internal-variable consistency, and the hysteresis-loop area defined by Equation (17) are checked sequentially.

Given $N_{FT}$, the parameter set, and the strain history, the incremental computational procedure is as follows: (1) read $E_{n}$, $f_{c,N}$, $\varepsilon_{c,N}$, and $r_{N}$, and calculate $d_{f}$; (2) update κ and η, and determine whether the current state corresponds to envelope loading, unloading, or reloading; (3) during envelope loading, calculate the stress using Equation (5), update $\varepsilon^{p}$ using Equation (7), and update $c_{m}$ and $d_{m}$ using Equations (9) and (10), respectively; (4) during unloading or reloading, hold the internal variables fixed and calculate the stress using Equation (14) or (15), respectively; (5) once the strain exceeds the historical maximum strain, return the response to the envelope according to Equation (16); and (6) when the cumulative damage induced by environmental exposure and mechanical loading is to be evaluated, calculate *D* using Equation (4) and assess the cyclic energy dissipation using Equation (17).

It should be noted that the number of quantities appearing in the complete formulation is larger than the number of independently calibrated parameters. The experimentally determined $E_{N}$, $f_{c,N}$, and $\varepsilon_{c,N}$ are treated as state inputs, whereas $d_{f}$, ω, $c_{m}$, $d_{m}$, D, and $W_{h}$ are derived state or diagnostic quantities and do not introduce additional fitting degrees of freedom. Accordingly, at each freeze–thaw exposure level, the independently calibrated parameter set consists of $r_{N}$, $A_{N}$, $B_{N}$, $n_{u,N}^{pre}$, $n_{u,N}^{post}$, $n_{r,N}^{pre}$, and $n_{r,N}^{post}$. Furthermore, owing to the hierarchical and piecewise structure of the formulation, these seven parameters do not act simultaneously on an individual response segment: $r_{N}$ governs the outer-envelope shape, $A_{N}$ and $B_{N}$ determine the residual-strain offset, and only one unloading or reloading exponent is active for a given cyclic branch.

### 2.9. Accuracy Metrics and Evidence Hierarchy

The fitting and reconstruction accuracy is jointly evaluated using the coefficient of determination *R*^2^ and the normalized root-mean-square error (NRMSE):

(21) $$R^{2}=1-\frac{\overset{n}{\underset{i=1}{\sum {\left(y_{i}^{cal}-y_{i}^{exp}\right)}^{2}}}\;}{\overset{n}{\underset{i=1}{\sum {\left(y_{i}^{exp}-{\bar{y}}^{exp}\right)}^{2}}}\;},NRMSE=\frac{\sqrt{\frac{1}{n}\underset{i}{\sum {\left(y_{i}^{cal}-y_{i}^{exp}\right)}^{2}}\;}}{y_{rel}}$$

where $y_{i}^{cal}$ is the calculated value at the *i*-th data point; $y_{i}^{exp}$ is the experimental value at the *i*-th data point; ${\bar{y}}^{exp}$ is the mean of the experimental values; *i* is the data-point index; *n* is the total number of data points. For the envelope stress, $y_{rel}$ is taken as the peak stress at the corresponding freeze–thaw exposure level; for the residual strain, $y_{rel}$ is taken as the peak strain; and for the normalized cyclic paths ($y_{rel}=1$). The coefficient of determination *R*^2^ quantifies the extent to which the overall variation in the data is captured, whereas the NRMSE measures the absolute error relative to the characteristic scale. For residual strain in particular, even a high *R*^2^ value may be accompanied by errors ranging from several tens to more than one hundred microstrain, which can produce a visible horizontal shift in the early hysteresis loops.

A two-level evidence chain is established in this study. Section 3 uses 36 monotonic compression curves of recycled coarse aggregate self-compacting concrete subjected to sulfate freeze–thaw exposure to evaluate $d_{f}$, $f_{c,N}$, $\varepsilon_{c,N}$, $r_{N}$, and the envelope structure. Section 4 uses cyclic compression data for ordinary concrete subjected to seawater freeze–thaw exposure to further evaluate $A_{N}$, $B_{N}$, the path exponents, the complete cyclic response, and the signed stiffness variable. The monotonic data examined in Section 3 cannot independently validate the residual strain or mechanical damage variables, while a high full-curve fitting accuracy in Section 4 does not establish the unique identifiability of the internal variables. Because the parameters at each freeze–thaw exposure level are calibrated using data from that same exposure level, the present results demonstrate parameter-identification and response-reconstruction capabilities rather than predictive capability across freeze–thaw exposure levels without recalibration.

## 3. Uniaxial Compressive Envelope Response and Parameter Evolution of RCASCC Subjected to Sulfate Freeze–Thaw Exposure

### 3.1. Data Source and Experimental Matrix

The uniaxial compression data for recycled coarse aggregate self-compacting concrete (RCASCC) subjected to sulfate freeze–thaw exposure reported by Zheng et al. [10] were adopted in this section to evaluate the monotonic-loading envelope submodel and the freeze–thaw-dependent state parameters established in Section 2. The experimental variables included the recycled coarse aggregate (RCA) replacement ratio, the mass fractions of Na_2_SO_4_ and MgSO_4_ in the exposure solution, and the number of freeze–thaw cycles. To avoid confusion between the letter “N” used in the group designation and the number of freeze–thaw cycles (Table 1), the latter is consistently denoted by $N_{FT}$. In the group notation, N0 and N5 denote Na_2_SO_4_ mass fractions of 0% and 5%, respectively, whereas M0 and M5 denote MgSO_4_ mass fractions of 0% and 5%, respectively.

Each of the six material–environment groups contained six freeze–thaw exposure levels, yielding a total of 36 representative monotonic compression curves. The peak stress $f_{c,N}$, peak strain $\varepsilon_{c,N}$, and static elastic modulus $E_{N}$ were obtained from the parameter tables reported in the original study, whereas the complete stress–strain coordinates were extracted by digitizing the published curves using Origin 2024 (OriginLab Corporation, Northampton, MA, USA). Because the publicly available data did not report the specimen-to-specimen variability or confidence intervals for replicate specimens under each condition, the analysis in this section is restricted to deterministic curve calibration and comparison of parameter-evolution trends; no statistical significance testing is performed.

Accordingly, differences among freeze–thaw exposure levels reported in the following sections should be interpreted as deterministic trends of the available representative curves rather than statistically significant differences among specimen populations.

Data verification revealed a systematic factor-of-ten discrepancy in the strain coordinates of the six digitized curves for the RC0-N0-M0 group relative to both the strain axis of the original figure and the tabulated peak strains. Because the same discrepancy was consistently observed at all freeze–thaw exposure levels within this group, the strain coordinates of the RC0-N0-M0 curves were uniformly multiplied by a factor of 10. Both the original and corrected coordinates were retained in the computational files to ensure traceability of the data-processing procedure. A small number of negative strain or stress values near the origin were attributed to image-digitization noise and were excluded from parameter identification and error evaluation.

### 3.2. Envelope Parameter Identification and Consistency Assessment

The monotonic stress–strain response was represented using the Mander-type envelope defined in Equation (5). The experimentally reported peak stress $f_{c,N}$ and peak strain $\varepsilon_{c,N}$ were held fixed, whereas the envelope-shape parameter $r_{N}$ was identified from the complete stress–strain curve by least-squares fitting according to Equation (18). The fitting accuracy was jointly evaluated using *R*^2^ and NRMSE, as defined in Equations (21) and (22), respectively. For the NRMSE calculation, the peak stress of the corresponding curve was used as the normalization reference.

The present analysis evaluates only the freeze–thaw pre-damage variable $d_{f}$, peak stress $f_{c,N}$, peak strain $\varepsilon_{c,N}$, and envelope-shape parameter $r_{N}$. Because the available monotonic data contain no unloading–reloading paths, the plastic residual strain $\varepsilon^{p}$, compaction variable $c_{m}$, and mechanical damage variable $d_{m}$ cannot be independently identified from the data considered in this section. Their identification and evaluation under cyclic loading are therefore addressed in Section 4.

If the experimentally reported static modulus $E_{N}$ is assumed to be identical to the initial tangent stiffness of the Mander envelope, Equation (6) can be rearranged to obtain the corresponding envelope-shape parameter:

(22) $$r_{N}^{\left(E\right)}=\frac{E_{N}\varepsilon_{c,N}}{E_{N}\varepsilon_{c,N}-f_{c,N}}=\frac{E_{N}}{E_{N}-f_{c,N}/\varepsilon_{c,N}}$$

where $r_{N}^{\left(E\right)}$ denotes the envelope-shape parameter inferred from the experimentally reported static modulus $E_{N}$ under the assumption $E_{cnv,N}=E_{N}$; $E_{cnv,N}$ is the equivalent initial tangent stiffness of the envelope curve at the specified freeze–thaw exposure level. Equation (22) is used solely as a consistency check between the experimentally reported static modulus and the equivalent initial tangent of the Mander envelope; it is not used for final parameter identification.

The final envelope representation therefore employs the independently calibrated $r_{N}$. The corresponding $E_{cnv,N}$, back-calculated from $r_{N}$ using Equation (6), represents only the equivalent initial tangent implied by the mathematical envelope and should not be interpreted as a replacement for the experimentally determined static modulus $E_{N}$. In particular, $E_{cnv,N}$ is not used to calculate the freeze–thaw pre-damage variable $d_{f}$.

### 3.3. Reconstruction of the Envelope Curves

**Figure 1:**
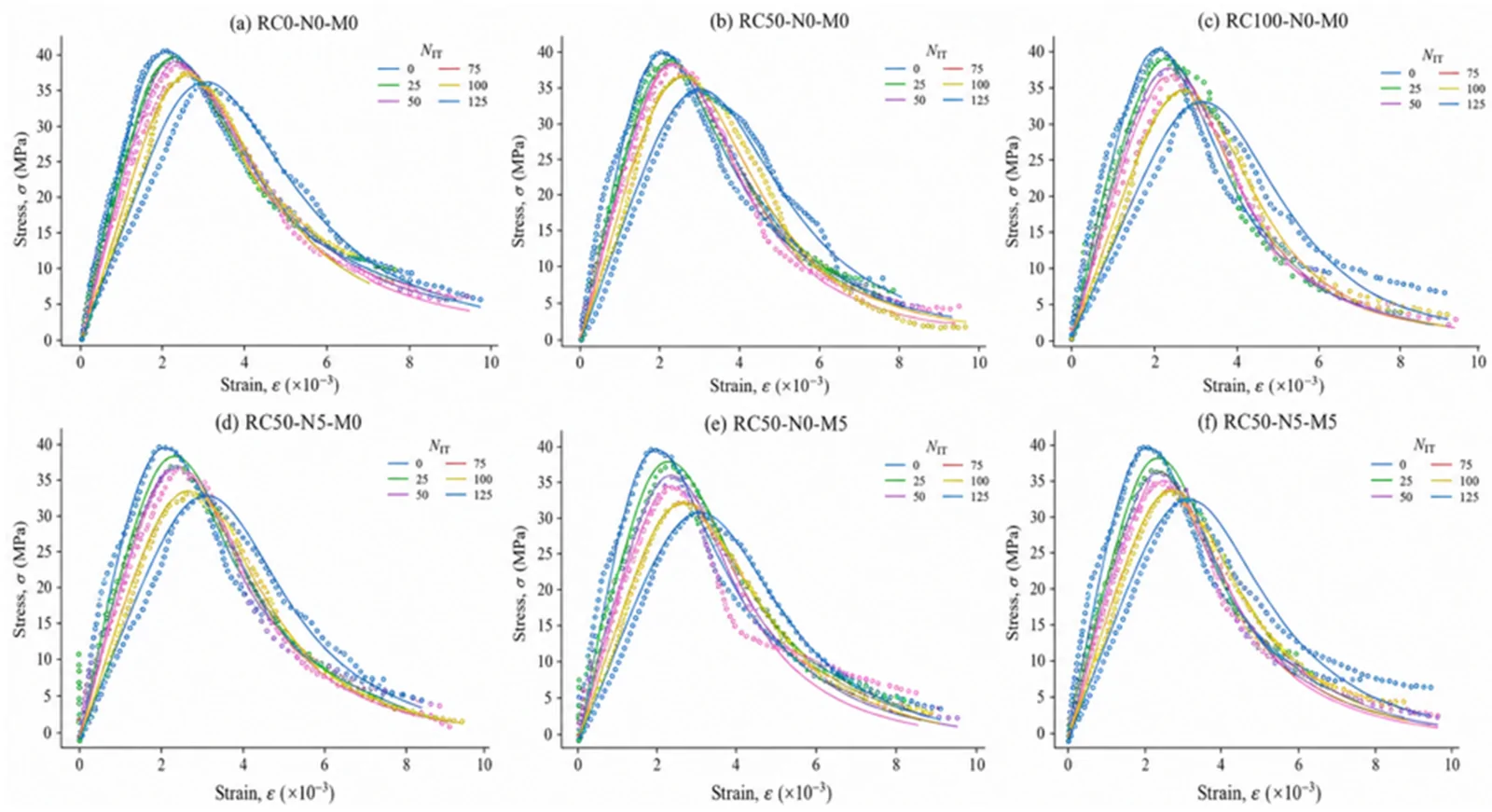
Experimental stress–strain curves and Mander-type envelope reconstructions of RCASCC with different material compositions under sulfate freeze–thaw environments: (**a**) RC0-N0-M0; (**b**) RC50-N0-M0; (**c**) RC100-N0-M0; (**d**) RC50-N5-M0; (**e**) RC50-N0-M5; and (**f**) RC50-N5-M5.

Figure 1 compares the experimental stress–strain curves with the corresponding model reconstructions for the six material–environment groups at different freeze–thaw exposure levels. The open symbols represent the experimental data, whereas the solid lines denote the Mander-type envelopes calculated using Equation (5) [2,3]. The proposed envelope submodel continuously reproduces the pre-peak ascending response, the location of the peak, and the post-peak softening branch, while also capturing the changes in stress level and strain scale associated with different freeze–thaw exposure levels.

Following independent calibration, the 36 envelope curves yielded a mean $R^{2}$ of 0.978, a median *R*^2^ of 0.984, and a mean NRMSE of 0.041. Across all curves, the minimum $R^{2}$ was 0.907, whereas the maximum NRMSE was 0.081. The lowest fitting accuracy occurred for the RC50-N0-M5 specimen after 75 freeze–thaw cycles; nevertheless, its $R^{2}$ remained above 0.90 (Table 2).

**Figure 2:**
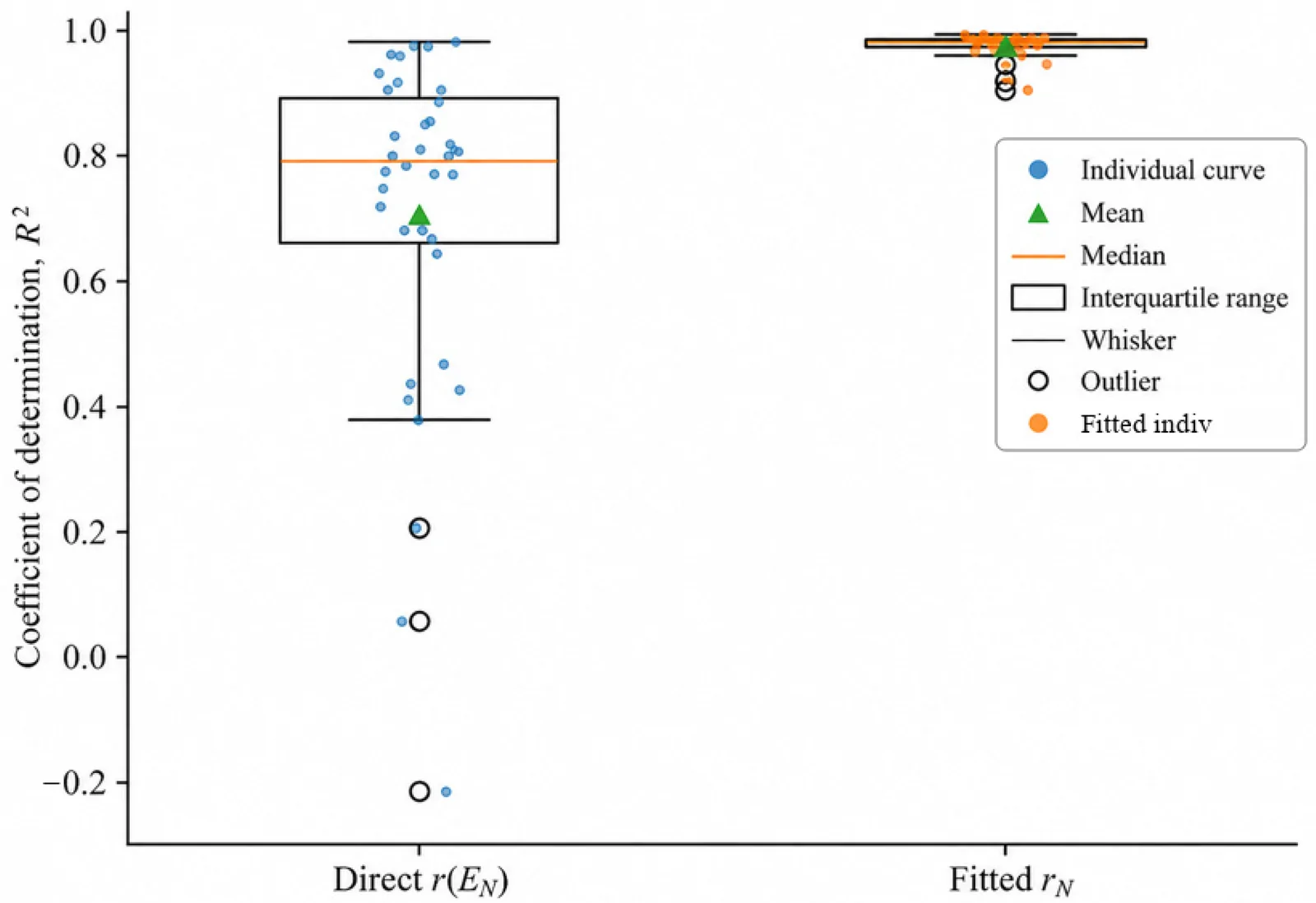
Distribution of *R*^2^ values obtained using the shape parameter inferred directly from the static elastic modulus and the independently calibrated $r_{N}$.

Figure 2 compares the distributions of *R*^2^ obtained using the shape parameter $r_{N}^{\left(E\right)}$ directly inferred from the experimentally reported static modulus $E_{N}$ and those obtained using the independently calibrated $r_{N}$. The direct-modulus-based approach yielded a mean *R*^2^ of only 0.705, with very low or even negative *R*^2^ values occurring for several curves at higher freeze–thaw exposure levels. In contrast, after independent calibration of $r_{N}$, all 36 curves achieved (*R*^2^ > 0.90). These results demonstrate that the experimentally reported static modulus $E_{N}$ and the equivalent initial tangent stiffness of the Mander-type envelope cannot be used interchangeably [10,12].

### 3.4. Evolution of the Principal State Parameters Under Freeze–Thaw Cycling

**Figure 3:**
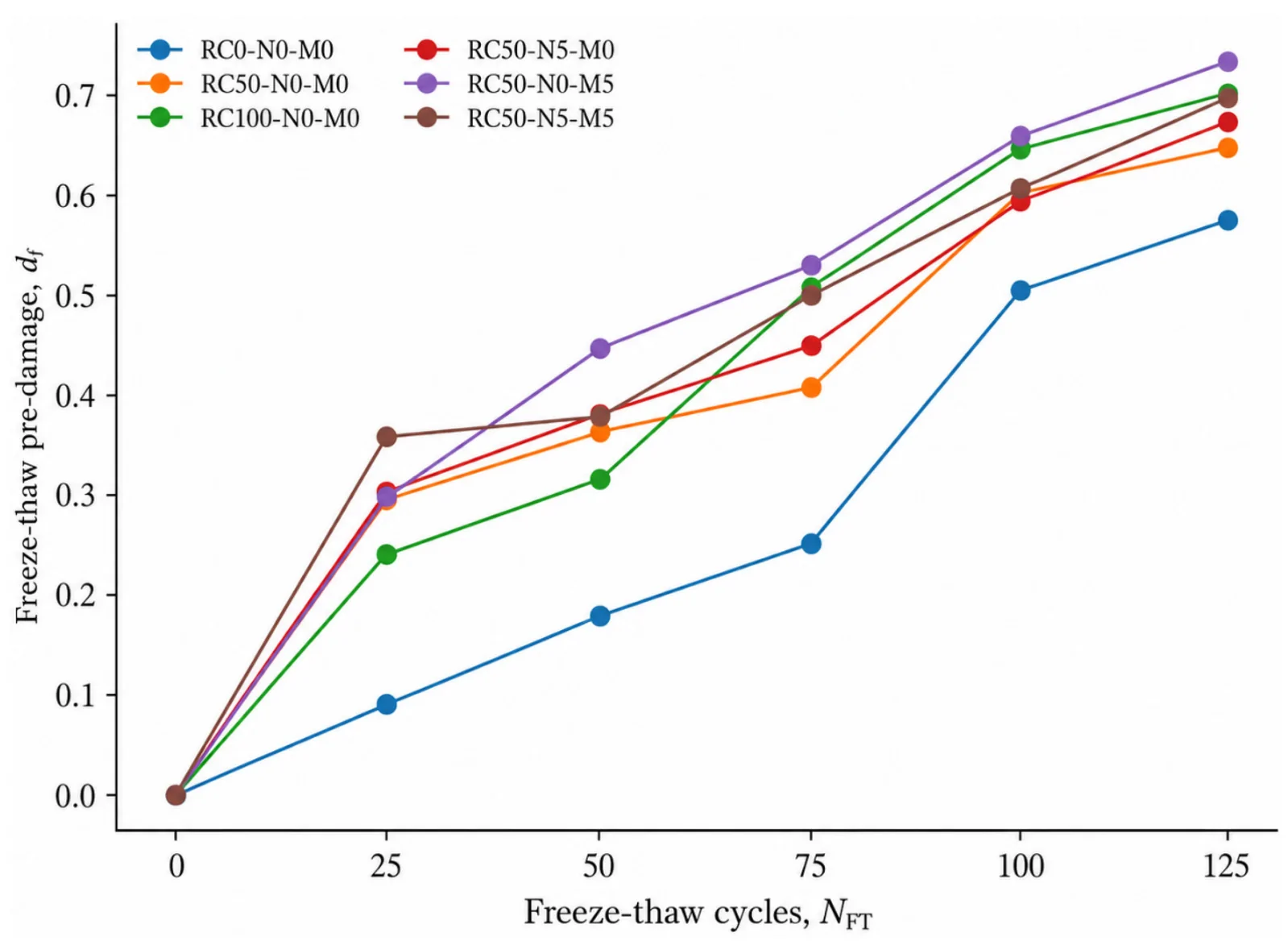
Freeze–thaw pre-damage defined based on the degradation of the static elastic modulus.

The freeze–thaw pre-damage variable $d_{f}$ was calculated from the retention ratio of the static elastic modulus for each group according to Equation (3). As shown in Figure 3, $d_{f}$ increased markedly with increasing $N_{FT}$. The rate of increase, however, was not uniform among the different material–environment groups. In particular, the RC0-N0-M0 control group exhibited a pronounced increase in $d_{f}$ between 75 and 100 freeze–thaw cycles, suggesting a stage of accelerated stiffness degradation after progressive accumulation of freeze–thaw-induced internal defects. In contrast, the RC50-N0-M5 and RC50-N5-M5 groups did not exhibit a comparably abrupt increase over the same interval, indicating that the onset and rate of stiffness degradation depend on both material composition and exposure environment. The sulfate-exposed mixtures may experience deterioration distributed differently over the freeze–thaw history because physical freeze–thaw damage interacts with the altered pore and interface characteristics of recycled aggregate concrete and sulfate-related effects. However, since the available dataset does not contain cycle-resolved microstructural observations or replicate-level variability, the specific mechanism responsible for these different stage-dependent trends cannot be uniquely determined. After 125 freeze–thaw cycles, the $d_{f}$ values of the RC0-N0-M0, RC50-N0-M0, and RC100-N0-M0 groups reached 0.573, 0.646, and 0.701, respectively. For the groups with an RCA replacement ratio of 50%, the corresponding $d_{f}$ values under the salt-free, Na_2_SO_4_, MgSO_4_, and combined-salt environments were 0.646, 0.672, 0.733, and 0.696, respectively.

The reductions in static elastic modulus after 125 freeze–thaw cycles ranged from 57.3% to 73.3% among the six groups and were consistently greater than the corresponding reductions in peak stress. This difference indicates that the static elastic modulus $E_{N}$ and peak stress $f_{c,N}$ exhibit different sensitivities to freeze–thaw exposure and therefore cannot be related through a single linear scaling relationship.

**Figure 4:**
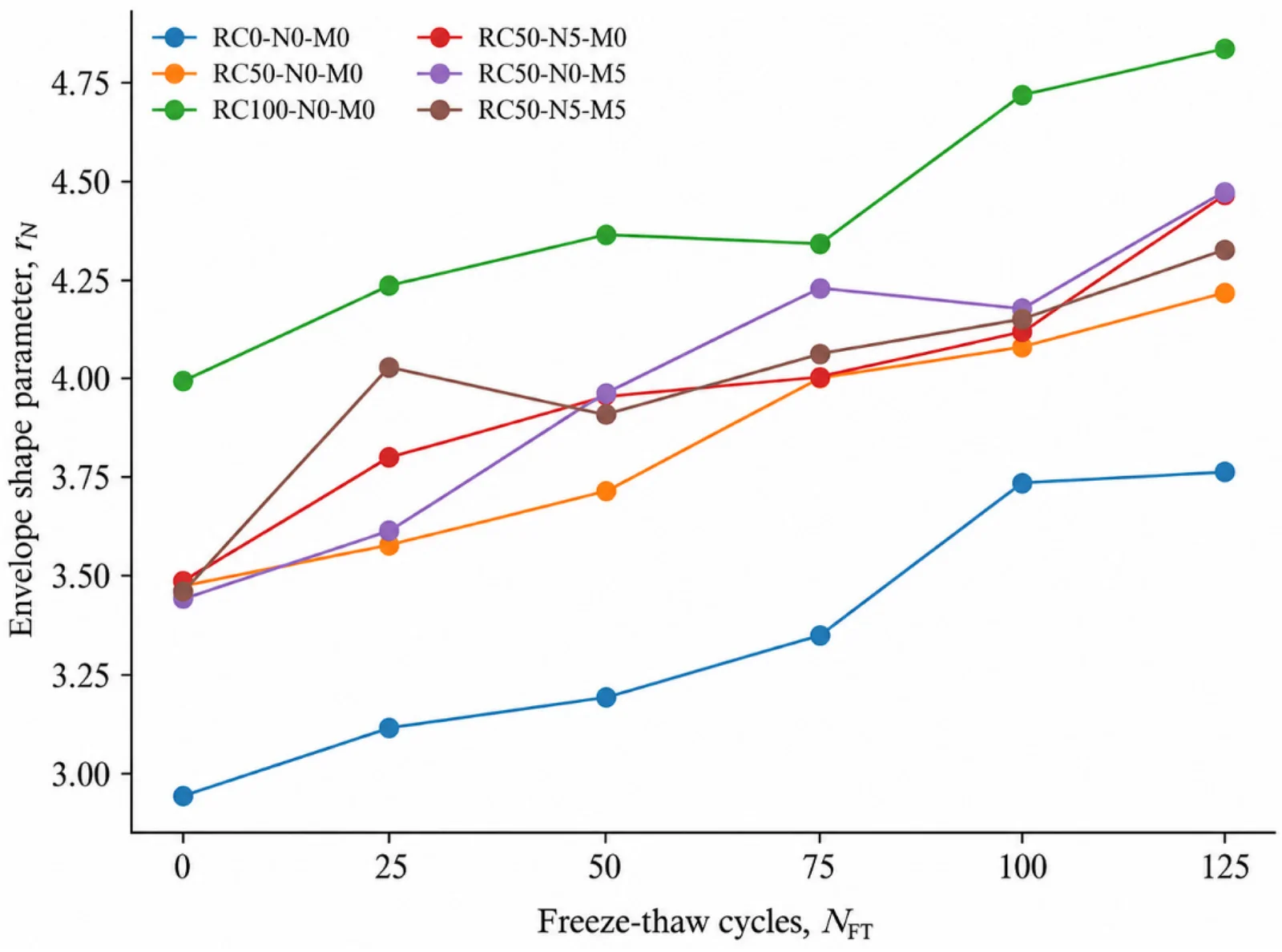
Evolution of the Mander-type envelope-shape parameter with the number of freeze–thaw cycles.

Figure 4 presents the independently identified envelope-shape parameter $r_{N}$. For all six groups, $r_{N}$ generally increased with increasing $N_{FT}$, although local fluctuations were observed. After 125 freeze–thaw cycles, $r_{N}$ increased by 21.0–29.7% relative to the corresponding unfrozen state. The largest increase occurred in the RC50-N0-M5 group, whereas the increases in the RC50-N0-M0 and RC100-N0-M0 groups were both approximately 21.0%. Because $r_{N}$ is simultaneously influenced by the curvature of the pre-peak branch, the width of the peak region, the post-peak descending response, and the weighting of the digitized data points during parameter identification, it is reported here solely as an envelope-shape parameter and is not assigned a unique physical interpretation in terms of brittleness or material homogeneity.

### 3.5. Comparison of Material Composition and Sulfate-Exposure Effects

Under salt-free conditions, the RC0, RC50, and RC100 groups provide a direct comparison of the effect of RCA replacement ratio. At $N_{FT}=0$, both the peak stress and static elastic modulus decreased with increasing RCA replacement ratio. After 125 freeze–thaw cycles, the reductions in peak stress for the three groups were 10.7%, 13.1%, and 18.1%, respectively, whereas the corresponding reductions in static elastic modulus were 57.3%, 64.6%, and 70.1% (Table 3). Because the available dataset contains only three RCA replacement ratios, namely 0%, 50%, and 100%, no higher-order continuous relationship between RCA replacement ratio and the response parameters is fitted in this section.

At a fixed RCA replacement ratio of 50%, the overall parameter changes after 125 freeze–thaw cycles exhibited the following trend: the MgSO_4_ single-salt group showed the greatest reductions in both peak stress and static elastic modulus, followed by the Na_2_SO_4_ single-salt group, whereas the combined-salt group exhibited an intermediate level of degradation. The macroscopic deterioration observed in the combined-salt environment was therefore not equivalent to a linear superposition of the responses obtained under the two single-salt environments. Because only two concentration levels, 0% and 5%, are available and no data on reaction products or microstructural evolution are provided, this section reports the observed non-additive response without assigning a specific chemical or interfacial mechanism. The underlying mechanisms are discussed further in Section 5.

As shown in Table 3, the four state parameters exhibit distinctly different rates of evolution. The static elastic modulus shows the greatest degree of degradation, whereas the reduction in peak stress is comparatively moderate; meanwhile, both the peak strain and $r_{N}$ generally increase with freeze–thaw cycling. These observations support the parameter framework established in Section 2, in which the freeze–thaw pre-damage variable is defined from $E_{N}$, while $f_{c,N}$, $\varepsilon_{c,N}$, and $r_{N}$ are retained as independent freeze–thaw-dependent state parameters. The material mechanisms underlying the different evolution patterns of these parameters, together with their consistency across different datasets, are discussed in detail in Section 5.

## 4. Cyclic Compressive Response and Damage Evolution of Ordinary Concrete Subjected to Seawater Freeze–Thaw Cycling

### 4.1. Experimental Data and Cyclic-Path Identification

The repeated uniaxial compression data for ordinary concrete subjected to seawater freeze–thaw cycling reported by Qiu et al. [13] were adopted in this section to evaluate the cyclic-response submodel, residual-strain relationship, and stiffness-related internal variables established in Section 2.

The tested material was conventional ready-mixed concrete. The cementitious materials consisted of Grade 42.5 ordinary Portland cement and Class II fly ash. The fine aggregate comprised natural sand and manufactured sand, whereas natural crushed stone was used as the coarse aggregate. The specimens measured 100 mm × 100 mm × 300 mm, and the water-to-binder ratio was 0.48. The numbers of rapid seawater freeze–thaw cycles, $N_{FT}$, were 0, 25, 50, 75, 100, and 125, and the compressive loading rate was 0.1 mm/min.

The cyclic stress–strain coordinates were obtained by digitizing the published curves and were retained in their original loading sequence. The datasets corresponding to the six freeze–thaw exposure levels contained 384, 412, 333, 280, 291, and 304 valid digitized coordinate points, respectively. The differences in point number arise from the amount of usable curve information recoverable from each published figure, including differences in visible path length and graphical resolution, and do not represent differences in the original experimental sampling frequency. The horizontal coordinate was consistently treated as dimensionless axial strain and is presented in the figures using a scale factor of ×10^−3^. The publicly available data consist of representative curves for each freeze–thaw exposure level and do not include the original high-frequency acquisition data from the testing machine, specimen-to-specimen variability among replicate tests, or confidence intervals. Accordingly, this section focuses on deterministic parameter identification and cyclic-path reconstruction, without statistical significance testing.

Cyclic reversal points were identified using local minima at low stress levels together with the maximum-strain point within the preceding loading interval. A cycle was regarded as valid when a distinct unloading onset, a subsequent low-stress reversal point, and the corresponding reloading branch could be identified from the digitized curve. On this basis, 7, 7, 7, 6, 7, and 8 valid cycles were identified for the six freeze–thaw exposure levels, respectively, yielding a total of 42 unloading stages. The variation in cycle number reflects the number of complete and distinguishable unloading–reloading loops recoverable from each published curve rather than a uniform cycle number imposed during the present analysis. Because the original high-frequency test records were unavailable, whether these differences originated from the experimental loading protocol or from the graphical representation of the published curves could not be further distinguished. Intermediate points generated during curve digitization were not treated as independent control points. Once the reloading path returned to the previously attained historical maximum strain, the response was switched back to the outer envelope.

### 4.2. Parameter Identification and Evaluation Procedures

The freeze–thaw pre-damage variable $d_{f}$ was calculated from the initial static stiffness *E_N_* according to Equation (3). The cyclic outer envelope was represented by Equation (5); the peak stress $f_{c,N}$ and peak strain $\varepsilon_{c,N}$ were determined from the corresponding experimental curve, whereas the envelope-shape parameter $r_{N}$ was identified according to Equation (18) (Table 4). The residual-strain parameters $A_{N}\;$ and $B_{N}$ were determined from the unloading onset points and the low-stress residual-strain points using Equation (19).

The unloading and reloading paths were described by Equations (13)–(16). According to whether $\eta ={\kappa /\varepsilon }_{c,N}$ satisfied η ≤ 1, or η > 1, the identified cycles were classified into pre-peak and post-peak regimes. The unloading-path exponent $n_{uN}^{s}$ and reloading-path exponent $n_{r,N}^{s}$ were then identified separately for the two regimes, where *s* denotes the pre-peak or post-peak regime. To prevent branches with a higher density of digitized points from dominating the optimization, each branch was assigned equal weight in the identification of the path parameters according to Equation (20).

The experimental unloading stiffness, signed stiffness variable *ω*, compaction variable $c_{m}$, and mechanical damage variable $d_{m}$ were evaluated according to Equations (8) and (10). The cumulative damage index *D* was then calculated from the freeze–thaw pre-damage and mechanical damage using Equation (4). The hysteretic energy dissipation $W_{h}$ was evaluated using Equation (17) together with numerical integration of the experimental branches. The envelope response, residual strain, and complete cyclic response were jointly assessed using $R^{2}$ and NRMSE. $W_{h}$ was used only as a diagnostic measure of the mechanical consistency of the cyclic paths and was not included in parameter calibration.

### 4.3. Basic Cyclic Response and Freeze–Thaw Pre-Damage

With increasing $N_{FT}$, the experimental cyclic curves exhibited decreases in peak stress and initial static stiffness, accompanied by overall increases in peak strain and residual strain. The digitized peak stress decreased from 36.643 MPa to 17.785 MPa, while the peak strain increased from 0.00191 to 0.00338. The initial static stiffness reported in the original study decreased from 32.0 GPa to 13.2 GPa, corresponding to an increase in $d_{f}$ from 0 to 0.588.

At $N_{FT}=50$, the digitized peak stress was 29.042 MPa, approximately 4.1% higher than the tabulated value of 27.9 MPa. At $N_{FT}=100$, the digitized peak strain was 0.00284, approximately 5.3% lower than the tabulated value of 0.0030. To maintain continuity of the envelope reconstruction at the peak point, the peak values extracted from the digitized curves were used for envelope identification, whereas $d_{f}$ continued to be calculated from the reported initial static stiffness $E_{N}$. These discrepancies and their implications for parameter uncertainty are discussed in Section 5.

### 4.4. Envelope Curve Fitting Results

**Figure 5:**
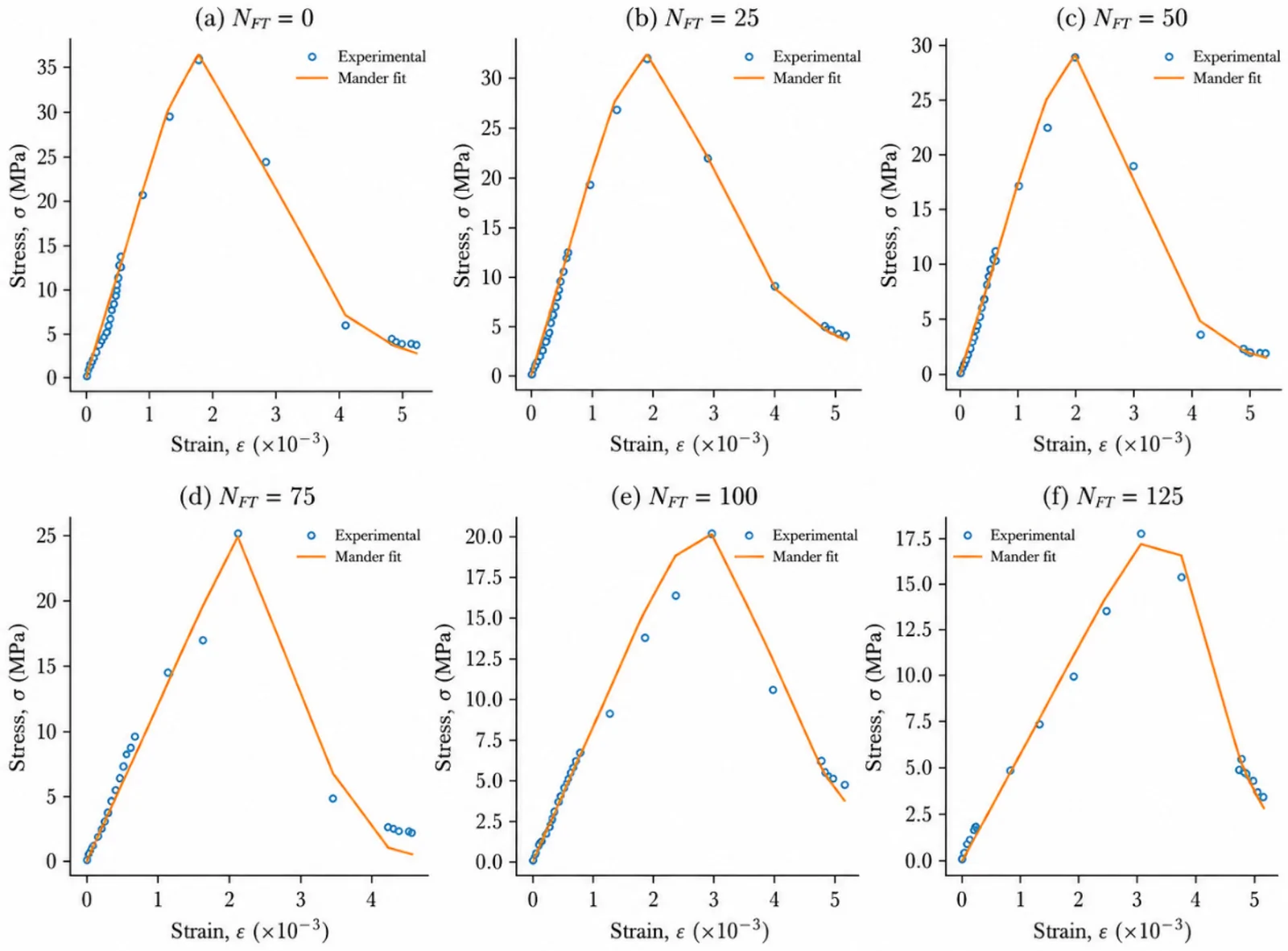
Comparison between experimental envelope data and the Mander-type model at different freeze–thaw exposure levels.

Figure 5 compares the experimental envelope data with the Mander-type model. Across the six freeze–thaw exposure levels, $R^{2}$ ranged from 0.958 to 0.989, with a mean value of 0.979, while NRMSE ranged from 0.024 to 0.046. The model continuously reproduces the pre-peak ascending branch, peak response, and post-peak softening. The curve at $N_{FT}=75$ exhibited a relatively abrupt post-peak stress drop and therefore yielded the lowest envelope $R^{2}$, although the value remained 0.958.

The independently identified $r_{N}$ values were 5.286, 4.892, 6.186, 10.779, 6.946, and 10.708 for $N_{FT}=0,25,50,75,100,\;and\;125$, respectively. The parameter $r_{N}$ did not vary strictly monotonically with $N_{FT}$. Accordingly, it is treated here only as the envelope-shape parameter associated with the corresponding freeze–thaw exposure level, and no continuous evolution function is proposed without cross-validation.

### 4.5. Plastic Residual Strain Fitting Results

**Figure 6:**
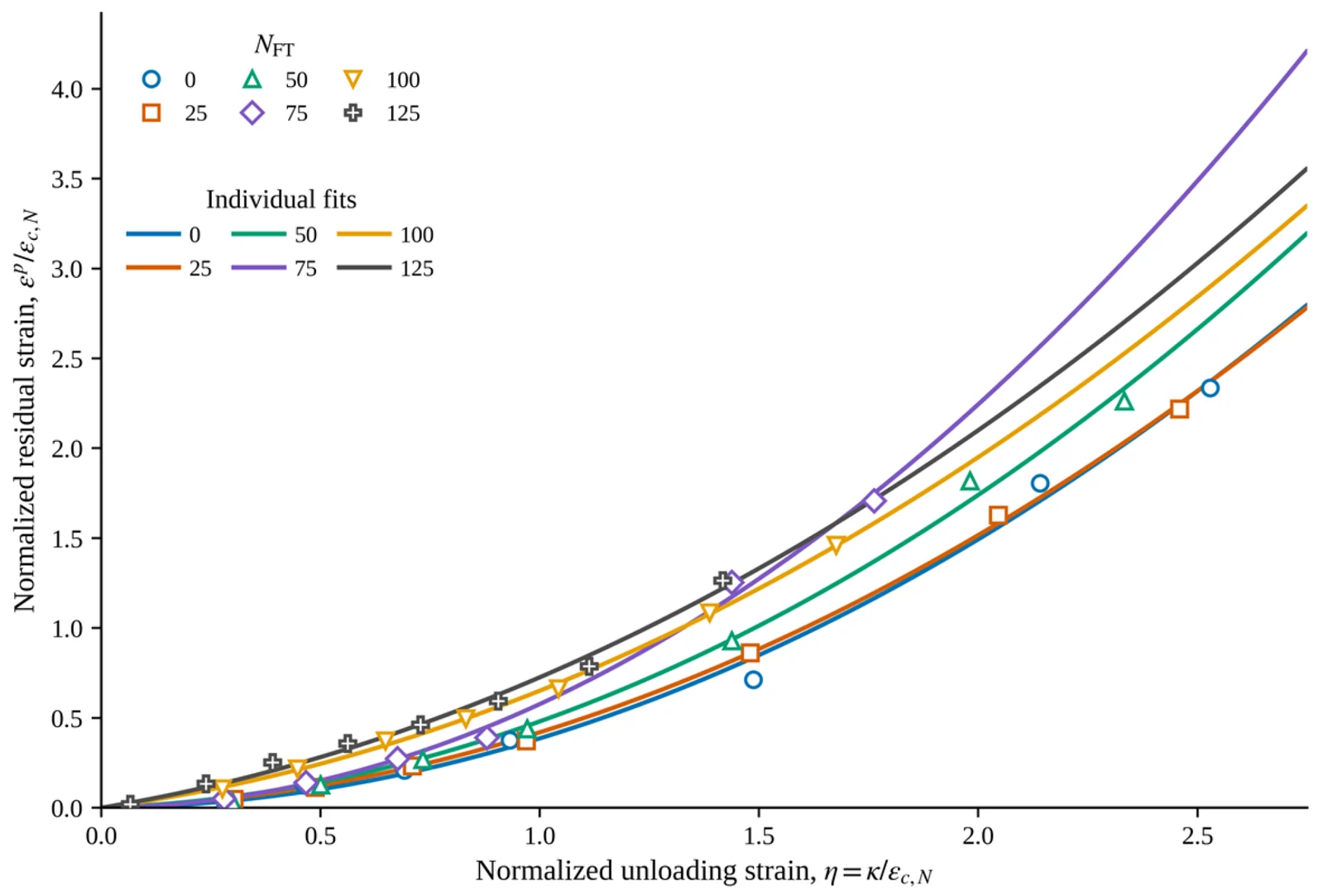
Experimental normalized residual strain and quadratic-model fitting results.

Figure 6 presents the experimental values of normalized residual strain together with the quadratic-model fits. Across the six freeze–thaw exposure levels, $R^{2}$ ranged from 0.991 to 0.999, while the absolute RMSE ranged from 43.5 to 123.7 με (as shown in Table 5). The proposed relationship captures the primary growth trend of residual strain with increasing η, although errors ranging from several tens to more than one hundred microstrain may still cause visible horizontal offsets in the smaller hysteresis loops.

### 4.6. Unloading–Reloading Path Parameters

**Figure 7:**
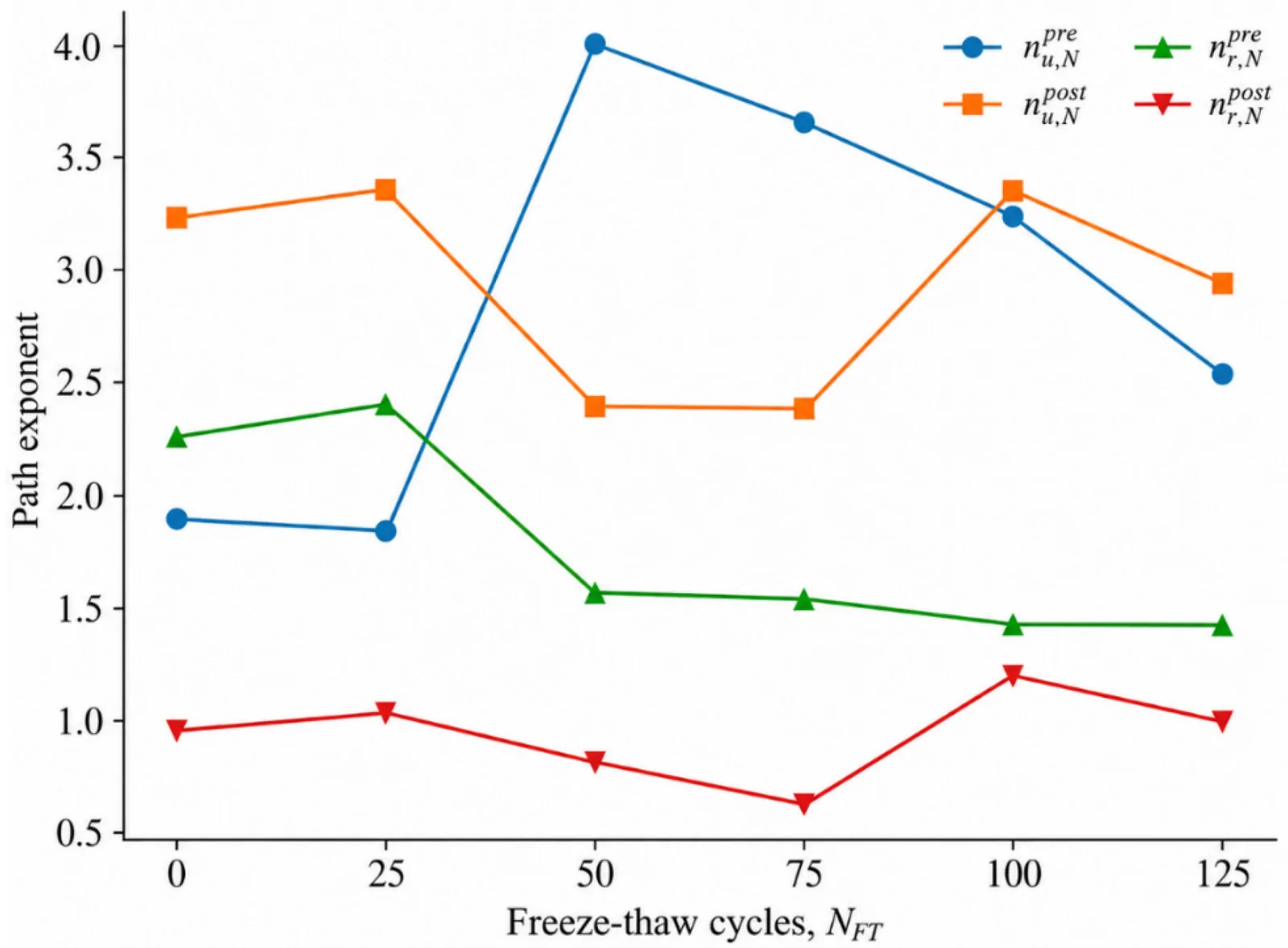
Pre-peak and post-peak unloading–reloading path parameters.

Figure 7 summarizes the path exponents identified for the pre-peak and post-peak regimes. The pre-peak unloading exponent ranged from 1.845 to 4.007, whereas the post-peak unloading exponent ranged from 2.386 to 3.356. The pre-peak reloading exponent ranged from 1.423 to 2.405, whereas the post-peak reloading exponent ranged from 0.626 to 1.197. None of the four parameter sets exhibited a strictly monotonic relationship with *N_FT_*.

By assigning independent path exponents to the pre-peak and post-peak regimes, the model can separately adjust the hysteresis-loop curvature in the relatively low-damage pre-peak stage and in the post-peak softening stage. The non-monotonic variation in these parameters with freeze–thaw exposure and the associated issue of parameter parsimony are discussed in Section 5. No micromechanical interpretation is inferred from these trends in the present section.

### 4.7. Reconstruction of the Complete Cyclic Response

**Figure 8:**
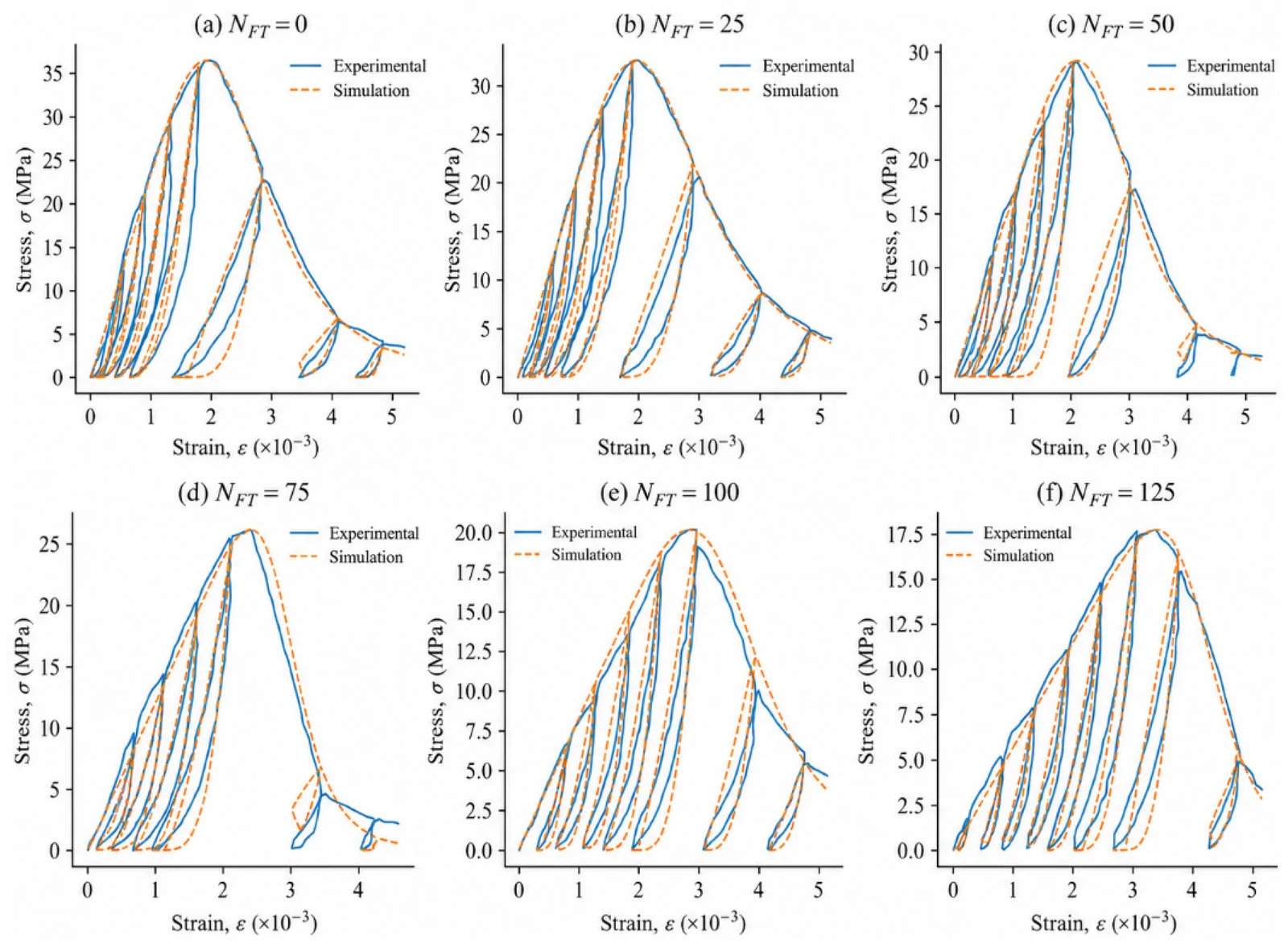
Comparison between the experimental cyclic stress–strain curves and complete-model reconstructions at different freeze–thaw exposure levels.

Figure 8 compares the experimental cyclic stress–strain curves with the responses reconstructed by the complete model. The $R^{2}$ values for $N_{FT}=0,25,50,75,100,$ and 125 were 0.908, 0.929, 0.953, 0.967, 0.928, and 0.960, respectively, with a mean $R^{2}$ of 0.941 and a mean NRMSE of 0.066. These statistics quantify within-exposure-level reconstruction accuracy after parameter identification using the same cyclic data and therefore do not constitute independent predictive validation. For $N_{FT}=0\;and\;25,$ the discrepancies were concentrated mainly in the early small hysteresis loops and portions of the reloading branches (as shown in Table 6). For $N_{FT}=50-125\;and\;25$, all $R^{2}$ values were at least 0.928.

The model simultaneously reproduces the degradation of peak stress, accumulation of residual strain, progressive rightward shift in the hysteresis loops along the strain axis, and post-peak softening response. The reported accuracy characterizes response-reconstruction capability after parameter identification using data from the same freeze–thaw exposure level; it should not be interpreted as predictive capability across different freeze–thaw exposure levels without recalibration.

### 4.8. Signed Stiffness Variable and Cumulative Damage

**Figure 9:**
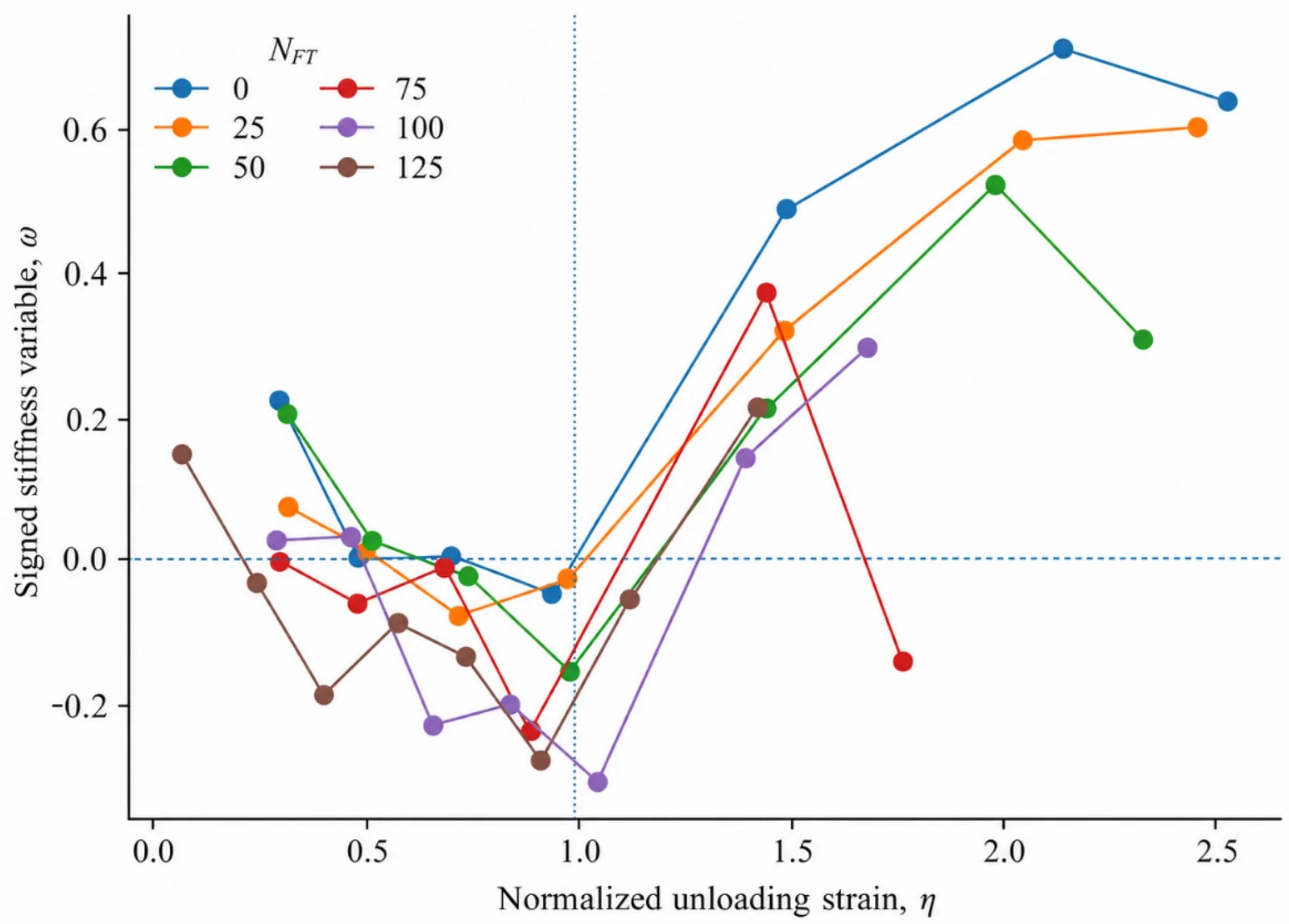
Variation in the signed stiffness variable ω with normalized unloading strain.

Figure 9 presents the signed stiffness variable ω for the 42 identified unloading stages. Among these stages, 19 satisfied ω<0 and 23 satisfied ω≥0, indicating that both stiffness enhancement and stiffness degradation relative to the post-freeze–thaw initial stiffness occurred at different freeze–thaw exposure levels and loading states. The minimum ω values for the six exposure levels were −0.047, −0.078, −0.154, −0.236, −0.308, and −0.277, respectively, corresponding to maximum compaction-variable values $c_{m}$ ranging from 0.047 to 0.308.

**Figure 10:**
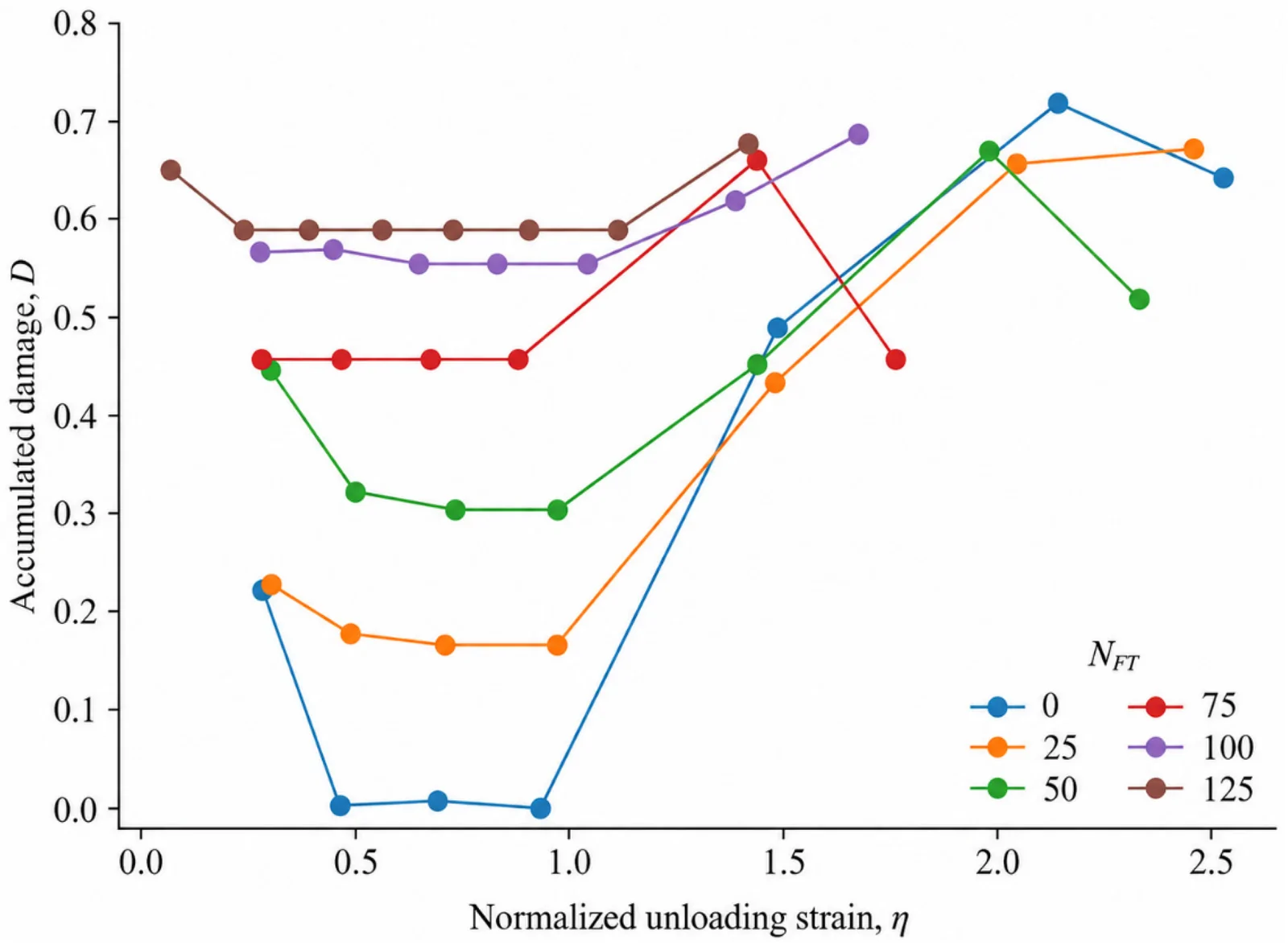
Evolution of cumulative damage with normalized unloading strain at different freeze–thaw exposure levels.

The maximum mechanical damage values $d_{m}$ obtained from the positive part of ω were 0.717, 0.604, 0.524, 0.372, 0.295, and 0.213 for $N_{FT}=0,25,50,75,100,\;and\;125,$ respectively. The decrease in the maximum ($d_{m}$) from 0.372 at 75 cycles to 0.295 at 100 cycles should not be interpreted as a reduction in the overall deterioration of the material. Over the same interval, the freeze–thaw pre-damage ($d_{f}$) increased from 0.456 to 0.553, while the minimum signed stiffness variable decreased from −0.236 to −0.308, indicating a lower post-freeze–thaw stiffness baseline together with more pronounced pre-peak stiffness enhancement relative to that baseline. Because ($d_{m}$) is defined relative to the corresponding post-freeze–thaw initial stiffness ($E_{N}$), an increase in environmental pre-damage may be accompanied by a smaller additional mechanically induced stiffness-loss ratio. Consistently, the maximum cumulative damage index (D) increased from 0.659 to 0.685 between 75 and 100 cycles. Therefore, the apparent reduction in ($d_{m}$) reflects the decomposition between environmental pre-damage and subsequent mechanical damage rather than material recovery. Combining these values with the freeze–thaw pre-damage *d_f_* according to Equation (4) yielded maximum cumulative damage indices D of 0.717, 0.670, 0.668, 0.659, 0.685, and 0.675, respectively. Because the identified cyclic histories differ in the number of loading stages, maximum normalized strain, and termination condition, the corresponding $D_{max}$ values are path-dependent and should not be directly compared as durability rankings among freeze–thaw exposure levels. A rigorous cross-level comparison would require a unified cyclic loading protocol and evaluation at the same normalized loading state. As shown in Figure 10, freeze–thaw pre-damage raises the baseline level of cumulative damage, whereas mechanically induced damage governs the subsequent increase in D with loading history at each freeze–thaw exposure level.

The calculated signed stiffness variable $\omega_{cal}$, back-calculated from the envelope stress and residual strain, was compared with the observed value $\omega_{obs}$ identified from the unloading stiffness. Across all 42 unloading stages, the overall *R*^2^ was approximately 0.553, substantially lower than the fitting accuracy obtained for the complete cyclic stress response. Accordingly, the signed-stiffness formulation should be regarded as a mathematical decomposition of the observed stiffness changes into enhancement and degradation components. It is consistent with the measured unloading-stiffness evolution but does not independently establish unique physical fractions of compaction and mechanical damage. Therefore, $c_{m}$, $d_{m}$, D and are used primarily for internal-state interpretation and summarization rather than as uniquely identified physical damage quantities. Therefore, $c_{m}$, $d_{m}$, and D are used primarily for interpretation and summarization of the internal state and should not be regarded as uniquely identified solely on the basis of the high $R^{2}$ values of the reconstructed stress–strain curves. The sources of this discrepancy are discussed in Section 5.

### 4.9. Hysteretic Dissipation and Mechanical Consistency of the Cyclic Paths

**Figure 11:**
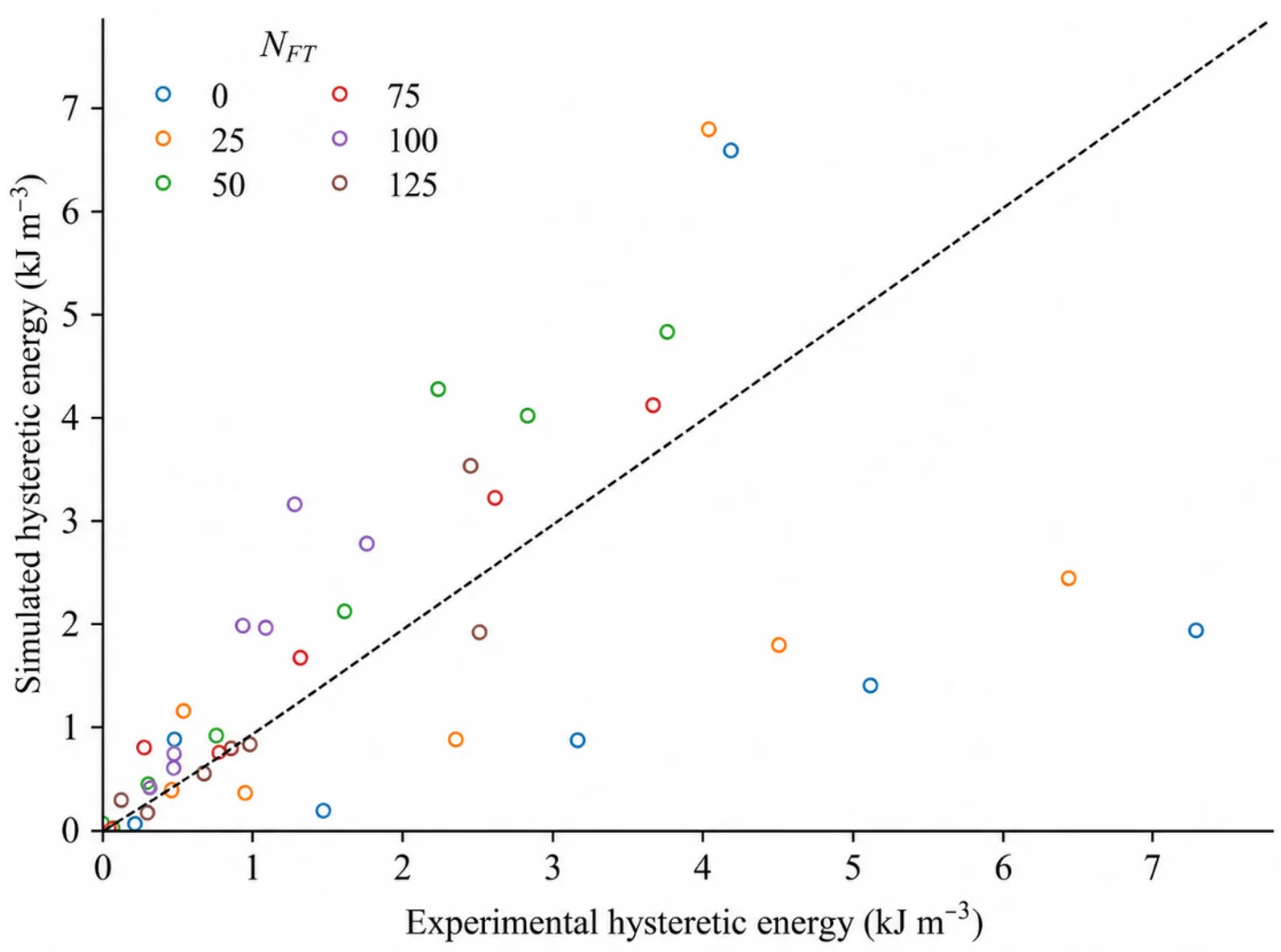
Comparison of experimental and reconstructed hysteretic dissipation magnitudes.

The hysteretic dissipation magnitude was calculated for all 42 cycles using Equation (17) together with numerical integration of the experimental and reconstructed branches. The model and experiment exhibited the same branch-area orientation in 37 of the 42 cycles. The disagreements occurred mainly in the first small cycle at $N_{FT}=0$ and 25 and in terminal hysteresis loops represented by relatively few digitized points. Figure 11 compares the experimental and reconstructed hysteretic dissipation magnitudes.

The overall $R^{2}$ for hysteretic dissipation magnitude was 0.207, with an RMSE of 1.582 kJ·m^−3^, substantially lower than the pointwise stress-reconstruction accuracy. The cumulative experimental dissipation values for $N_{FT}=0,25,50,75,100,\;and\;125$ were 21.96, 19.31, 11.56, 8.73, 6.39, and 8.05 kJ·m^−3^, respectively, whereas the corresponding model values were 11.91, 13.76, 16.60, 10.55, 11.57, and 8.09 kJ·m^−3^ (as shown in Table 7). Because the number of cyclic loading stages and the imposed loading levels were not identical among the different freeze–thaw exposure levels, these cumulative values are used only for diagnostic comparison within the present loading paths and are not treated as independent durability indicators [12,16,17,18,19].

## 5. Discussion

Section 2 established a one-dimensional cyclic damage–plasticity framework for concrete subjected to prior freeze–thaw damage. Section 3 and Section 4 provided hierarchical evaluations using monotonic compression data for sulfate freeze–thaw RCASCC and cyclic compression data for seawater freeze–thaw ordinary concrete, respectively. Rather than repeating parameter values and fitted curves, this section discusses the model conclusions jointly supported by the two datasets, the differences among evidence levels, possible interpretations of the material and environmental effects, and the boundaries that should govern further use of the model for prediction and structural analysis.

### 5.1. Cross-Dataset Evidence Chain and Model Positioning

Section 3 contains six material–environment groups and 36 monotonic curves. The Mander-type envelope achieved a mean $R^{2}$ of 0.978 and a mean NRMSE of 0.041. Section 4 contains six seawater freeze–thaw exposure levels and 42 identified cycles; the mean envelope $R^{2}$ was 0.979, and the mean $R^{2}$ for the complete cyclic response was 0.941. Although the two datasets differ in material type, exposure medium, and loading path, both can be described using the freeze–thaw-dependent state parameters and the Mander-type outer envelope established in Section 2.

The primary implication of this result is structural applicability of the model architecture: the same organization of state variables and computational framework can be applied to different material–environment datasets after recalibration. This does not imply transferability of the calibrated parameter values from sulfate freeze–thaw RCASCC to seawater freeze–thaw ordinary concrete, nor does it imply that the cyclic submodel has been independently validated for RCASCC or sulfate environments. Section 3 provides evidence for the monotonic envelope, whereas Section 4 additionally provides evidence for residual strain, cyclic-path curvature, stiffness-related internal variables, and hysteretic dissipation.

The primary implication of this result is structural transferability of the model: the same organization of state variables and the same computational framework can be applied to different datasets after parameter recalibration. It does not imply that a single parameter set can be transferred directly from sulfate freeze–thaw RCASCC to seawater freeze–thaw ordinary concrete, nor does it imply that the cyclic submodel has been validated for RCASCC or sulfate environments. Section 3 provides evidence only for the monotonic envelope, whereas Section 4 additionally provides evidence for residual strain, cyclic-path curvature, stiffness-related internal variables, and hysteretic dissipation.

**Figure 12:**
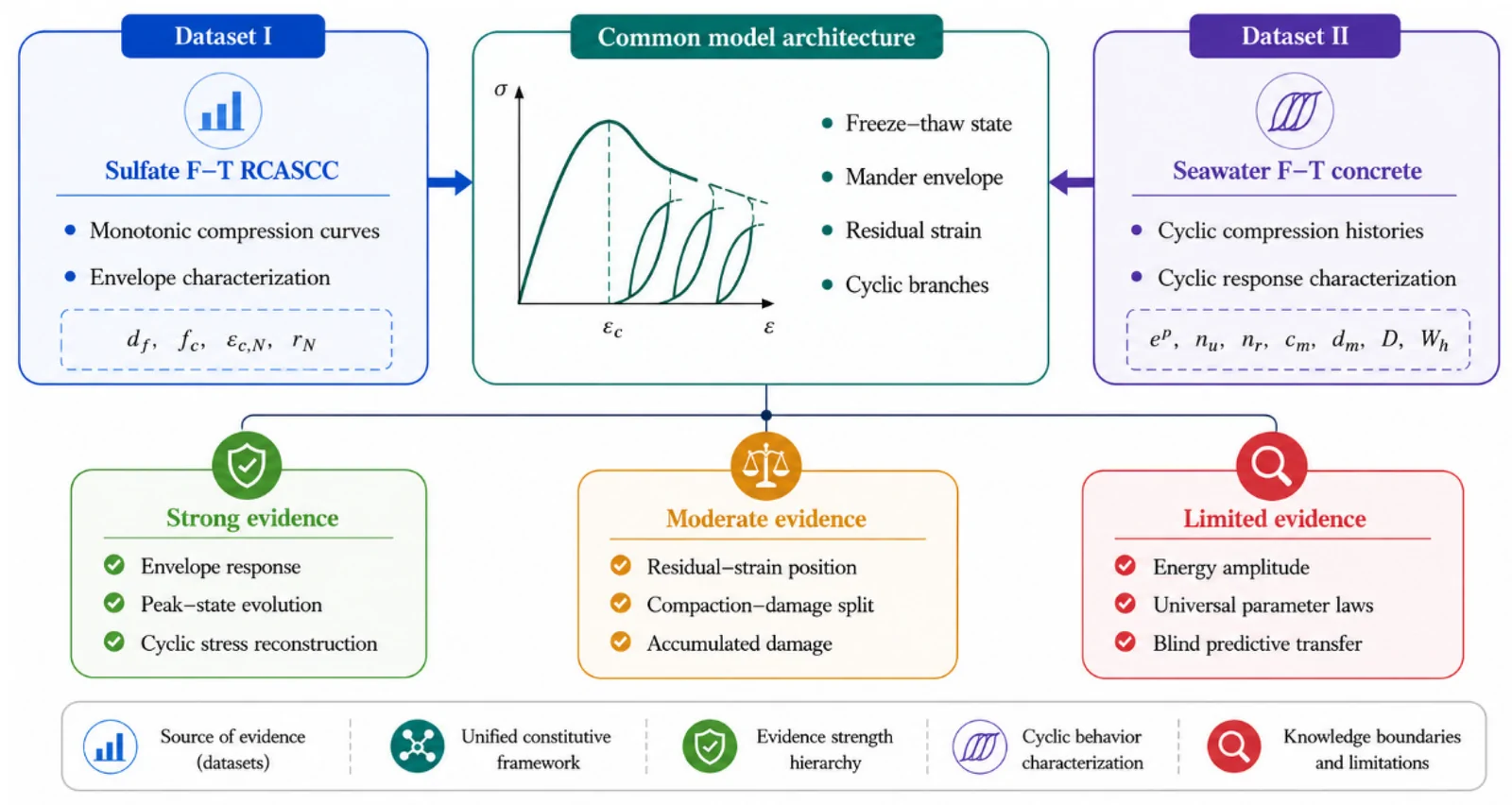
Cross-dataset evidence hierarchy of the constitutive framework.

Accordingly, the present model should be positioned as a framework with structural consistency across material–environment datasets, while its parameters remain dependent on material type, exposure medium, and freeze–thaw exposure level. Predictive transferability can only be claimed after sufficient accuracy is demonstrated for an independent material or freeze–thaw exposure level without recalibration of the principal parameters (Figure 12 and Table 8).

### 5.2. Parameter Hierarchy and State Characterization of Freeze–Thaw Deterioration

Both datasets show that freeze–thaw exposure affects different macroscopic parameters to markedly different extents. After 125 cycles, the static elastic modulus loss of sulfate freeze–thaw RCASCC ranged from 57.3% to 73.3%, whereas the peak-stress loss was only 10.7–21.9%. For seawater freeze–thaw ordinary concrete, the initial static stiffness decreased from 32.0 GPa to 13.2 GPa, the peak stress decreased from 36.643 MPa to 17.785 MPa, and the peak strain increased from 0.00191 to 0.00338.

These results indicate that a single strength-retention ratio cannot represent the full range of freeze–thaw-induced changes in stiffness and deformation scale. Defining $d_{f}$ from $E_{N}$ in Section 2 effectively uses a stiffness measure that is sensitive to distributed microcracking and interfacial degradation as the environmental-state baseline. However, $d_{f}$ describes only the initial degradation at the stiffness level and cannot simultaneously replace $f_{c,N}$, $\varepsilon_{c,N}$, or $r_{N}$. These four parameter classes represent the initial stiffness, peak load-bearing capacity, peak location, and normalized envelope shape, respectively. Because they evolve at different rates, they should be retained as related but non-equivalent state variables.

In Section 3, $r_{N}$ generally increased with freeze–thaw cycling, whereas in Section 4 it exhibited pronounced fluctuations across exposure levels. This difference further indicates that $r_{N}$ should not be interpreted directly as a universal damage index. Its value is influenced simultaneously by material composition, pre-peak curvature, peak-region width, post-peak descending response, and the weighting of digitized data points, making it more appropriately interpreted as a dataset-specific envelope-shape parameter.

The rightward shift in the peak strain should likewise not be interpreted directly as an increase in ductility. An increase in peak strain may simultaneously contain contributions from pore closure, interfacial slip, stable microcrack propagation, and damage accumulation. Moreover, the cyclic dissipation results in Section 4 did not show a uniform increase with peak strain, and the loading sequences differed among freeze–thaw exposure levels. Ductility or energy-absorption capacity must therefore be evaluated using independent measures such as post-peak deformation, curve area, or structural deformation capacity.

### 5.3. Effects of RCA Replacement Ratio and Sulfate Environment

For the salt-free groups in Section 3, both the peak-stress loss and static elastic-modulus loss increased with increasing RCA replacement ratio. This trend is consistent with material characteristics associated with adhered old mortar, pre-existing cracks, and the increased number of interfacial transition zones in recycled aggregate concrete. Previous microhardness studies have indicated that old interfacial transition zones and pre-existing cracks in recycled aggregate concrete may form relatively weak stress-transfer paths [25]. During freeze–thaw cycling, deformation in the interfacial transition zone is typically greater than that in the aggregate and may be associated with crack formation [26].

However, the dataset used in this study contains no measurements of water absorption, pore structure, interfacial microhardness, or crack statistics. The interpretation that adhered old mortar and multiple interfacial structures increase freeze–thaw sensitivity is therefore only consistent with existing studies and cannot be regarded as a micromechanical mechanism directly demonstrated by the present curves. The amount of adhered old mortar, the quality of the parent concrete, and the pre-saturation state of RCA from different sources may all modify this trend. In addition, the three available replacement ratios of 0%, 50%, and 100% are insufficient to establish a continuous optimum range.

At an RCA replacement ratio of 50%, the MgSO_4_ single-salt group exhibited the greatest losses in strength and static elastic modulus after 125 freeze–thaw cycles, followed by the Na_2_SO_4_ single-salt group, while the combined-salt group showed an intermediate level of deterioration. Previous studies of coupled freeze–thaw and sulfate exposure indicate that such environments involve the combined effects of pore-water freezing, salt-crystallization pressure, and sulfate chemical reactions, and that macroscopic deterioration under MgSO_4_ exposure may exceed that under Na_2_SO_4_ exposure [27,28,29]. The adhered old mortar and new interfacial zones in recycled concrete may also constitute regions that are sensitive to sulfate transport and reaction [28].

These studies provide a plausible physicochemical context for the present results but cannot substitute for the XRD, SEM, thermal-analysis, or ion-distribution evidence absent from the current dataset. In particular, the deterioration of the combined-salt group was not equal to the linear sum of the two single-salt groups and should therefore be described only as a macroscopic non-additive response. Because only two concentration levels, 0% and 5%, are available and a complete factorial design is absent, the current evidence can establish neither synergism nor antagonism or ion-competition mechanisms.

### 5.4. Physical Interpretation of the Transition from Pre-Peak Compaction to Post-Peak Damage

Among the 42 unloading stages in Section 4, 19 satisfied ω < 0 and 23 satisfied ω ≥ 0. If a conventional non-negative damage variable were imposed strictly, cases with ω < 0 would have to be truncated or misinterpreted as “negative damage.” By introducing $c_{m}$ in Section 2, stiffness enhancement relative to the post-freeze–thaw initial stiffness $E_{N}$ can be separated from mechanical damage, thereby preserving the experimental observation that the pre-peak unloading stiffness may exceed the initial stiffness.

ω < 0 is consistent with pore compaction, closure of initial microcracks, progressive formation of aggregate-contact networks, and enhanced interfacial friction. At higher levels of freeze–thaw deterioration, the initial stiffness is lower and the population of closable defects may be larger; the general increase in $c_{m,max}$ observed in Section 4 is therefore physically plausible. Nevertheless, $c_{m}$ is an operational variable constructed from the difference between two macroscopic stiffness measures. Its value is also affected by the measurement interval used for $E_{N}$, the unloading point, identification of the residual point, and digitization error. Therefore, $c_{m}$ cannot be interpreted directly as the fraction of closed microcracks.

After ω becomes positive in the post-peak regime, $d_{m}$ represents the reduction in unloading stiffness relative to $E_{N}$ and can be associated with dominant-crack propagation, interfacial debonding, frictional sliding, and localized crushing. The decrease in $d_{m,max}$ with increasing $N_{FT}$ does not imply that the mechanically induced damage of freeze–thaw-exposed specimens becomes less severe. Rather, $d_{f}$ has already raised the initial damage baseline, and the maximum normalized loading level and termination condition differ among the curves. Correspondingly, $D_{max}$ varies non-monotonically between 0.659 and 0.717 and should not be used directly to rank freeze–thaw durability. A stricter comparison would require the same η, the same level of stress reduction, or a unified loading protocol.

### 5.5. Residual Strain, Cyclic Paths, and Hysteretic Dissipation

The quadratic residual-strain relationship achieved $R^{2}$ values of 0.991–0.999 across the six seawater freeze–thaw exposure levels, indicating that the historical maximum strain captures the principal growth trend of residual deformation. However, the absolute RMSE remained 43.5–123.7 με, which is not negligible for the early small hysteresis loops. Because $R^{2}$ is strongly influenced by the overall data range, the residual-strain model should be evaluated together with absolute error rather than judged solely from a high $R^{2}$ value.

Using independent pre-peak and post-peak path exponents increased the mean $R^{2}$ of the complete cyclic stress response to 0.941, indicating that the power-law branches can reproduce the principal unloading–reloading curvature. However, none of the four path-exponent classes varies strictly monotonically with $N_{FT}$. This behavior suggests that pre-peak compaction, post-peak crack propagation, frictional slip, and curve-digitization error act simultaneously and cannot yet be summarized adequately by a single function of freeze–thaw cycles.

Hysteretic dissipation is more sensitive to local path errors than pointwise stress reconstruction. In Section 4, the model preserved the same branch-area orientation as the experiment in 37 of 42 cycles, but the $R^{2}$ for dissipation magnitude was only 0.207. Contributing factors include the use of a single pre-peak or post-peak exponent for each unloading or reloading family, the absence of loop pinching, slip-initiation thresholds, and cycle-by-cycle stiffness evolution, amplification of small coordinate errors in the area of small loops, and differences in the number of cycles and loading levels among freeze–thaw exposure levels.

Accordingly, the current path model prioritizes reconstruction of the stress history and should not be regarded as a high-accuracy energy model. Future parameter identification may impose branch ordering and non-negative dissipation as constraints and may introduce a moderately weighted energy term in addition to the stress-residual objective function. However, the dissipation fit should not be improved mechanically by introducing an excessive number of cycle-specific parameters.

### 5.6. Difference Between Curve-Reconstruction Accuracy and Internal-Variable Identifiability

A central distinction in the present study is that high reconstruction accuracy of the external stress response does not imply that the internal variables have been uniquely and accurately identified. The mean envelope $R^{2}$ values in Section 3 and Section 4 are both close to 0.98, and the mean $R^{2}$ for the complete cyclic response is 0.941. In comparison, the overall $R^{2}$ between $\omega_{cal}$ and $\omega_{obs}$ is approximately 0.553, and the $R^{2}$ for hysteretic-dissipation magnitude is only 0.207.

This evidence gradient has a clear origin. Stress is a directly digitized observable; residual strain requires identification of the low-stress intersection; unloading stiffness is calculated as the ratio of a stress difference to a relatively small strain difference and is therefore more sensitive to endpoint errors; the damage variables depend additionally on the definition of $E_{N}$; and hysteretic dissipation integrates the area between two branches, thereby accumulating errors over an entire curve segment. Uncertainty is therefore progressively amplified as the quantities of interest move from directly observed stress to derivatives, ratios, and integrals.

This progressive loss of agreement should therefore be interpreted as an evidence hierarchy rather than as equivalent levels of constitutive validation. The directly digitized stress–strain response provides the strongest evidence for the response-reconstruction capability of the framework. Residual strain and stiffness-related quantities provide a weaker level of evidence because their identification depends on selected curve features and coordinate differences, whereas hysteretic dissipation is still more sensitive because digitization errors accumulate through path integration. In addition, the absence of original machine-acquisition records, replicate-level data, and confidence intervals prevents separation of digitization uncertainty from specimen-to-specimen experimental variability. The present values of $R^{2}$ for the signed stiffness variable and hysteretic dissipation should therefore not be interpreted as validation of uniquely determined micromechanical internal variables, but as quantitative indicators of the current limits of their interpretation from the available data.

Accordingly, $c_{m}$, $d_{m}$ and D are operational material-point state variables inferred from macroscopic stiffness changes and should not be interpreted as uniquely measured fractions of microcrack closure or micromechanical damage. The envelope-driven, internal-variable-interpreted one-dimensional phenomenological framework defined in Section 2 was deliberately adopted to avoid treating a high curve $R^{2}$ as sufficient validation of a complete thermodynamic plasticity model.

### 5.7. Parameter Parsimony, Transferability, and Predictive Boundaries

Although the complete formulation contains several state and diagnostic quantities, only seven parameters are independently calibrated at each freeze–thaw exposure level: $r_{N}$, $A_{N}$, $B_{N}$, $n_{u,N}^{pre}$, $n_{u,N}^{post}$, $n_{r,N}^{pre}$, and $n_{r,N}^{post}$. The experimentally determined peak and stiffness quantities are state inputs, whereas $d_{f}$, ω, $c_{m}$, $d_{m}$, D, and $W_{h}$ are derived quantities. Moreover, because the formulation is hierarchical and piecewise, the complete parameter set is not simultaneously active at an individual stress–strain point.

To quantify the influence of the calibrated parameters, a normalized one-at-a-time perturbation analysis was conducted. For each parameter p, the remaining parameters were held fixed and p was varied symmetrically by ±10%. The normalized sensitivity index was defined as

(23) $$S_{p}=\frac{{∥q\left[p\left(1+0.10\right)\right]-q\left[p\left(1-0.10\right)\right]∥}_{2}}{0.20∥q\left(p\right){∥}_{2}}$$

where q denotes the normalized response directly governed by the corresponding parameter. For $r_{N}$, the normalized envelope stress was evaluated separately over the ascending (0≤x≤1) and post-peak (1≤x≤2) regimes. For $A_{N}$ and $B_{N}$, the normalized residual strain was evaluated over 0≤η≤1.5, whereas the four cyclic-path exponents were evaluated over 0≤χ≤1.

The sensitivity results reveal a clear hierarchical pattern rather than uniform sensitivity to all parameters. The mean sensitivity of $r_{N}$ was only 0.105 on the ascending branch but increased to 0.756 in the post-peak regime, confirming that this parameter primarily controls the descending-envelope shape. For the residual-strain relationship, the mean sensitivities of $A_{N}$ and $B_{N}$ were 0.730 and 0.275, respectively, indicating that $A_{N}$ provides the dominant contribution to the nonlinear horizontal migration of the hysteresis loops, whereas $B_{N}$ acts mainly as a secondary correction. The corresponding mean sensitivities of $n_{u,N}^{pre}$, $n_{u,N}^{post}$, $n_{r,N}^{pre}$, and $n_{r,N}^{post}$ were 0.597, 0.605, 0.549, and 0.458, respectively (Table 9). The same ranking was retained when perturbation amplitudes of 5% and 20% were used, indicating that the conclusions are not specific to the selected 10% perturbation.

Parameter parsimony was further examined by analytically reducing the cyclic-path formulation. If all four path exponents are set equal to unity, the unloading and reloading branches become linear and coincident. According to Equation (17), this reduction necessarily gives $W_{h}=0$, and therefore cannot represent the experimentally observed hysteresis. A less restrictive two-exponent model was also examined by imposing a common unloading exponent for the pre-peak and post-peak regimes and, separately, a common reloading exponent. The shared exponents were selected to minimize the normalized $L_{2}$ deviation from the calibrated four-exponent branch family. The resulting mean normalized branch-shape deviation was 0.143, with values ranging from 0.037 to 0.203 among the six freeze–thaw exposure levels. At $N_{FT}=0$, 25, 50, and 75, the corresponding deviations were approximately 19.8%, 20.3%, 15.8%, and 18.6%, respectively. Thus, collapsing the pre-peak and post-peak path parameters into a single unloading–reloading pair produces a non-negligible loss of branch-shape information for most of the examined exposure states.

These results do not establish the four-exponent formulation as a unique or universally optimal parameterization. Rather, they demonstrate that the additional regime separation has a measurable effect on the cyclic response and is not merely an inactive increase in the number of adjustable parameters. The present formulation should therefore be regarded as a phenomenological compromise between path fidelity and parameter parsimony. More aggressive parameter compression should be evaluated against independent cyclic datasets before being adopted for predictive applications.

From a cross-dataset perspective, the present study demonstrates that the Mander-type envelope and hierarchical organization of state variables can be applied, after recalibration, to both sulfate freeze–thaw RCASCC and seawater freeze–thaw ordinary concrete. What remains unproven is whether the calibrated parameter values or their freeze–thaw evolution laws can be transferred blindly to other materials or exposure states.

### 5.8. Data Uncertainty and Study Limitations

In addition to the issues listed in Table 10, the two datasets differ in material composition, freeze–thaw medium, specimen characteristics, and loading protocol. These differences are useful for examining whether the model structure has a nontrivial range of applicability, but they also prevent direct comparison of the absolute material-parameter values. Cross-dataset conclusions in the present study should therefore be restricted to structural consistency of the framework rather than consistency of absolute parameter values. Because replicate-level measurements are unavailable, the present deterministic analysis cannot separate digitization uncertainty from intrinsic specimen-to-specimen variability or provide statistical confidence intervals for the identified parameters. Within a broader uncertainty-quantification framework, these limitations involve distinct but interacting sources associated with experimental data, parameter identification, and model-form representation. The present study does not attempt to assign probability distributions to these sources or propagate them into predictive intervals; such an analysis would require richer replicate-level and original acquisition data than are currently available.

### 5.9. Engineering Implications and Future Research

From an engineering-modeling perspective, the proposed framework provides a layered means of separating environmental action from the subsequent mechanical response. The freeze–thaw pre-damage variable $d_{f}$ can be used to initialize the material stiffness state; $f_{c,N}$, $\varepsilon_{c,N}$, and $r_{N}$ define the monotonic envelope; and $\varepsilon^{p}$ together with the path exponents further describe the cyclic response. This structure is suitable as a prototype for a one-dimensional material-point model, parameter-sensitivity analysis, or a user-material subroutine.

At its present stage, the framework is intended for constitutive interpretation and response reconstruction at the uniaxial material-point level, rather than for direct deployment as a general structural finite-element material model. Direct application of the model in structural finite-element analysis nevertheless requires at least three additional developments. First, post-peak softening must be regularized using fracture energy, nonlocal formulations, or viscous regularization to avoid mesh dependence. Second, distinct tensile and compressive damage evolution laws and crack-closure rules are required. Third, multiaxial yield surfaces, plastic flow, and consistent constitutive updates are needed before the one-dimensional response can be extended to general stress states.

Future experiments should adopt unified cyclic sequences and maximum normalized strains, acquire the original load–displacement records from the testing machine, and simultaneously characterize ultrasonic response, pore structure, digital image correlation, acoustic emission, or fracture-surface features [30,31]. Such measurements would allow the environmental-state interpretation of $d_{f}$, the defect-closure interpretation of $c_{m}$, and the crack-propagation interpretation of $d_{m}$ to be examined independently. In terms of model validation, leave-one-freeze–thaw-exposure-level prediction should be prioritized before testing the parameter-evolution relationships against independent materials and exposure media.

Recent studies have also demonstrated the applicability of explicit cyclic constitutive modeling to concrete after freeze–thaw exposure [32]. For the cyclic paths, energy-consistency constraints, cycle-by-cycle stiffness evolution, or a minimal pinching parameter may be introduced while retaining a parsimonious parameterization. Multi-objective calibration could then be used to control stress error, residual-strain error, and hysteresis-area error simultaneously. Any such extension should demonstrate its benefit through external validation rather than solely through an increase in fitting accuracy on the calibration data.

## 6. Conclusions

A one-dimensional damage–plasticity constitutive framework was developed for freeze–thaw-damaged concrete subjected to a sequential “freeze–thaw exposure followed by mechanical loading” path. The framework was evaluated using monotonic compression data of recycled coarse aggregate self-compacting concrete (RCASCC) subjected to sulfate freeze–thaw cycles and cyclic compression data of ordinary concrete subjected to seawater freeze–thaw cycles. The main conclusions are as follows:

1. Analysis of 36 monotonic compression curves of sulfate freeze–thaw-damaged RCASCC showed that the experimentally measured static elastic modulus should be distinguished from the equivalent initial tangent stiffness of the Mander envelope. Independent identification of the envelope-shape parameter increased the mean $R^{2}$ from 0.705 to 0.978, with a mean NRMSE of 0.041, supporting the separate characterization of freeze–thaw pre-damage and envelope shape.
2. Freeze–thaw cycling caused markedly greater degradation in static elastic modulus (57.3–73.3%) than in peak stress (10.7–21.9%), while peak strain and the envelope-shape parameter generally increased. These different sensitivities indicate that strength loss alone is insufficient to characterize the mechanical state after freeze–thaw damage.
3. For seawater freeze–thaw-damaged ordinary concrete, the quadratic residual-strain relationship achieved $R^{2}$ values of 0.991–0.999, while the complete cyclic responses yielded a mean $R^{2}$ of 0.941 and a mean NRMSE of 0.066. Among 42 unloading stages, 19 exhibited stiffness enhancement and 23 exhibited stiffness degradation, supporting the distinction between pre-peak compaction and post-peak mechanical damage.
4. The same hierarchical constitutive architecture was applied to the two datasets after dataset-specific and exposure-level-specific parameter identification. Freeze–thaw pre-damage characterizes the initial stiffness state, whereas the peak-response, envelope-shape, residual-strain, and cyclic-path parameters describe different levels of the subsequent mechanical response. The results therefore demonstrate structural consistency and calibrated response-reconstruction capability of the framework across the examined datasets. They do not establish transferability of the calibrated parameter values or independent predictive capability for untested freeze–thaw exposure levels. Further leave-one-exposure-level or external-data validation is required before such predictive capability can be established.

## Figures and Tables

**Table 1:** Experimental matrix of the RCASCC uniaxial compression data.

Group	RCA Replacement Ratio (%)	Na_2_SO_4_/%	MgSO_4_/%	$N_{FT}$
RC0-N0-M0	0	0	0	0, 25, 50, 75, 100, 125
RC50-N0-M0	50	0	0	0, 25, 50, 75, 100, 125
RC100-N0-M0	100	0	0	0, 25, 50, 75, 100, 125
RC50-N5-M0	50	5	0	0, 25, 50, 75, 100, 125
RC50-N0-M5	50	0	5	0, 25, 50, 75, 100, 125
RC50-N5-M5	50	5	5	0, 25, 50, 75, 100, 125

**Table 2:** Envelope-fitting accuracy and range of shape parameters for each material–environment group.

Group	Mean *R*^2^	Minimum *R*^2^	Mean NRMSE	*r*_0_	*r*_125_
RC0-N0-M0	0.984	0.969	0.034	2.951	3.762
RC50-N0-M0	0.986	0.979	0.036	3.482	4.215
RC100-N0-M0	0.978	0.947	0.042	3.993	4.830
RC50-N5-M0	0.973	0.920	0.044	3.493	4.463
RC50-N0-M5	0.968	0.907	0.048	3.450	4.473
RC50-N5-M5	0.978	0.947	0.041	3.465	4.324

Note: $r_{0}$ and $r_{125}$ denote the independently identified envelope-shape parameters at $N_{FT}=0$ and $N_{FT}=125$, respectively.

**Table 3:** Changes in the principal state parameters after 125 freeze–thaw cycles relative to the corresponding ($N_{FT}=0$) state.

Group	Peak-Stress Reduction (%)	Peak-Strain Increase (%)	Elastic-Modulus Reduction (%)	$d_{f}(N_{FT}=125)$	Increase in ($r_{N}$) (%)
RC0-N0-M0	10.7	45.8	57.3	0.573	27.5
RC50-N0-M0	13.1	44.7	64.6	0.646	21.0
RC100-N0-M0	18.1	47.2	70.1	0.701	21.0
RC50-N5-M0	16.3	47.1	67.2	0.672	27.7
RC50-N0-M5	21.9	54.3	73.3	0.733	29.7
RC50-N5-M5	17.3	50.5	69.6	0.696	24.8

**Table 4:** Experimental groups, basic mechanical parameters, and freeze–thaw pre-damage.

$N_{FT}$	Valid Data Points	Identified Cycles	$f_{c,N}$ (MPa)	$\varepsilon_{c,N}$	$E_{N}$ (GPa)	$d_{f}$	$r_{N}$
0	384	7	36.643	0.00191	32.0	0.000	5.286
25	412	7	32.571	0.00196	26.7	0.166	4.892
50	333	7	29.042	0.00210	22.3	0.303	6.186
75	280	6	26.339	0.00240	17.4	0.456	10.779
100	291	7	20.227	0.00284	14.3	0.553	6.946
125	304	8	17.785	0.00338	13.2	0.588	10.708

**Table 5:** Envelope and residual-strain parameters and fitting accuracy.

$N_{FT}$	$r_{N}$	$A_{N}$	$B_{N}$	$R_{env}^{2}$	${NRMSE}_{env}$	$R_{\varepsilon p}^{2}$	${RMSE}_{\varepsilon p}\left(\mu \varepsilon \right)$
25	4.892	0.3386	0.0811	0.987	0.027	0.999	43.5
50	6.186	0.3898	0.0906	0.989	0.024	0.996	110.0
75	10.779	0.5455	0.0312	0.958	0.046	0.995	107.2
100	6.946	0.3240	0.3271	0.972	0.039	0.999	45.0
125	10.708	0.3245	0.4011	0.988	0.031	0.991	120.8

**Table 6:** Unloading–reloading parameters and reconstruction accuracy of the complete cyclic response.

$N_{FT}$	$n_{u}$, $N_{pre}$	$n_{u}$, $N_{post}$	$n_{r}$, $N_{pre}$	$n_{r}$, $N_{post}$	$R_{cycle}^{2}$	${NRMSE}_{cycle}$
0	1.898	3.229	2.260	0.953	0.908	0.082
25	1.845	3.356	2.405	1.033	0.929	0.074
50	4.007	2.399	1.571	0.813	0.953	0.059
75	3.663	2.386	1.542	0.626	0.967	0.052
100	3.233	3.350	1.427	1.197	0.928	0.072
125	2.540	2.942	1.423	0.990	0.960	0.056

**Table 7:** Summary of stiffness state, cumulative damage, and hysteretic dissipation.

$N_{FT}$	$\omega_{min}$	$c_{m,max}$	$d_{m,max}$	$D_{max}$	$\Sigma W_{exp}$ (kJ·m^−3^)	$\Sigma W_{sim}$ (kJ·m^−3^)	Branch-Area Sign Agreement
0	−0.047	0.047	0.717	0.717	21.96	11.91	6/7
25	−0.078	0.078	0.604	0.670	19.31	13.76	6/7
50	−0.154	0.154	0.524	0.668	11.56	16.60	6/7
75	−0.236	0.236	0.372	0.659	8.73	10.55	5/6
100	−0.308	0.308	0.295	0.685	6.39	11.57	7/7
125	−0.277	0.277	0.213	0.675	8.05	8.09	7/8

**Table 8:** Model components established in Section 2 and the strength of evidence provided by Section 3 and Section 4.

Model Component	Evidence from Section 3	Evidence from Section 4	Current Evidence Level	Supported Conclusions
Freeze–thaw pre-damage $d_{f}$	Six group-specific static-modulus sequences	Six seawater freeze–thaw exposure levels	Relatively strong	Can serve as an environmental-state indicator at the stiffness level
Mander-type envelope	36 monotonic curves; mean $R^{2}$ = 0.978	Six cyclic outer envelopes; mean $R^{2}$ = 0.979	Strong	The envelope structure shows cross-dataset applicability
Residual strain $\varepsilon^{p}$	No cyclic data; not evaluable	$R^{2}=0.991-0.999$	Relatively strong, but limited to within-level calibration	Can describe the horizontal position of the hysteresis loops
Unloading–reloading paths	No cyclic data; not evaluable	Complete cyclic response: mean $R^{2}$ = 0.941	Relatively strong	Can reconstruct the principal cyclic stress response
$c_{m}$, $d_{m}$, and D	Not evaluable	Consistency of ω: $R^{2}$ ≈ 0.553	Moderate	Suitable for internal-state interpretation; not uniquely identifiable
Hysteretic dissipation $W_{h}$	Not evaluable	Amplitude: $R^{2}$ = 0.207; branch-area direction consistent in 37/42 cycles	Limited	Suitable only as a mechanical diagnostic
Cross-exposure prediction	Blind validation not performed	Parameters re-identified at each exposure level	Not established	Universal predictive capability cannot be claimed

**Table 9:** Normalized sensitivity of the independently calibrated model parameters.

Parameter	Response Component	Mean Sensitivity	Range
$r_{N}$	Ascending envelope	0.105	0.074–0.136
$r_{N}$	Post-peak envelope	0.756	0.662–0.807
$A_{N}$	Residual-strain evolution	0.730	0.488–0.954
$B_{N}$	Residual-strain evolution	0.275	0.047–0.520
$n_{u,N}^{pre}$	Pre-peak unloading	0.597	0.558–0.631
$n_{u,N}^{post}$	Post-peak unloading	0.605	0.587–0.618
$n_{r,N}^{pre}$	Pre-peak reloading	0.549	0.525–0.587
$n_{r,N}^{post}$	Post-peak reloading	0.458	0.394–0.500

**Table 10:** Major sources of uncertainty, their effects, and recommended treatment.

Source of Uncertainty	Effect on the Present Conclusions	Recommended Treatment
Curve digitization rather than original machine-acquisition data	Peak values, residual points, unloading stiffness, and energy are all affected by coordinate error	Obtain original sampling data or report sensitivity intervals associated with digitization
Representative curves with no replicate specimens	Within-group variability and statistical significance cannot be evaluated	Add replicate specimens and confidence intervals
A fixed 10-fold strain correction for one group in Section 3	Introduces additional data-traceability and scale uncertainty	Explicitly report the correction factor and document the basis for the correction in the main text
A 4–5% difference between digitized and tabulated peak values in Section 4	Affects the envelope peak and some identified parameters	Report the tabulated and curve-calibrated values separately
Different numbers of cycles and maximum loading levels among exposure states	$D_{max}$ and cumulative $W_{h}$ cannot be compared directly as durability rankings	Use a unified cyclic protocol and compare at common η levels
The same data are used for calibration and evaluation	Reported accuracy reflects reconstruction rather than independent prediction	Perform leave-one-exposure-level validation or external-data validation
One-dimensional local softening model	Subject to mesh dependence and unable to represent multiaxial behavior or tension–compression transitions	Introduce fracture-energy regularization and extend the formulation to three dimensions

## Data Availability

The original contributions presented in this study are included in the article. Further inquiries can be directed to the corresponding author.
